# Mass-Loaded Metamaterials Based on Polymer Matrix with Static–Dynamic Decoupling for Pressure-Resistant Broadband Underwater Sound Absorption

**DOI:** 10.3390/polym18172109

**Published:** 2026-08-30

**Authors:** Lin Liu, Yang Wang, Shengsen Xu, Haibin Yang, Jie Zhong, Caiqiong Liang, Jihong Wen

**Affiliations:** 1 College of Intelligence Science and Technology, National University of Defense Technology, Changsha 410073, China; 2 National Key Laboratory of Equipment State Sensing and Smart Support, National University of Defense Technology, Changsha 410073, China

**Keywords:** mass-loaded metamaterial (MLM), modulus–density decoupling, sound velocity reduction, pressure-resistant, underwater sound absorption

## Abstract

Elastic metamaterials have been widely applied in elastic wave manipulation. Nevertheless, their performance is inherently restricted by two intrinsic coupling mechanisms: the modulus and density follow a power-law scaling relationship under static conditions, and the wave velocity is positively correlated with the modulus under dynamic conditions. To overcome these limitations, this study proposes a type of elastic metamaterial based on 3D-printed polymers, namely mass-loaded metamaterial (MLM). The structure is constructed by embedding discrete mass blocks into a lattice frame. The proposed design realizes the dual decoupling of modulus from density and wave velocity. The elastic modulus of the structure can be maximally increased by 307% while keeping the wave velocity constant; additionally, a 35% reduction in wave velocity can be achieved without deteriorating the elastic modulus. This distinctive decoupling effect possesses great application value in the field of underwater sound absorption. The structure can simultaneously exhibit high modulus and low wave velocity characteristics, which improves sound absorption performance and guarantees mechanical stability under high hydrostatic pressure.

## 1. Introduction

The macroscopic properties of materials, such as mechanical, acoustic, and thermal characteristics, are closely related to their microstructures [1,2]. Tailoring the configurations of microstructures has long been a conventional strategy to achieve specific material functionalities [3]. Over the past two decades, the emergence of metamaterials, which are artificially engineered structures that use tailored arrangements of macroscopic unit cells rather than atomic-scale constituents, has enabled extraordinary physical properties across numerous disciplines [4,5,6,7,8,9]. Driven by the rapid advancement in additive manufacturing [10,11], mechanical metamaterials can be readily fabricated from conventional materials, with target performance realized efficiently through structural design [12,13]. As a major branch, elastic metamaterials have received intensive research interest [14,15]. Current studies primarily focus on two major goals: one is to achieve enhanced static properties, such as high specific stiffness [16,17] and tunable Poisson’s ratio [18,19]; the other is to realize dynamic wave modulation [20], demonstrated through bandgap characteristics [21], negative effective properties [22,23,24], and wave steering/focusing effects [25,26,27]. Nevertheless, the coupling between static and dynamic responses in elastic metamaterials has received limited attention, representing a critical gap in the pursuit of multifunctional design.

**Figure 1:**
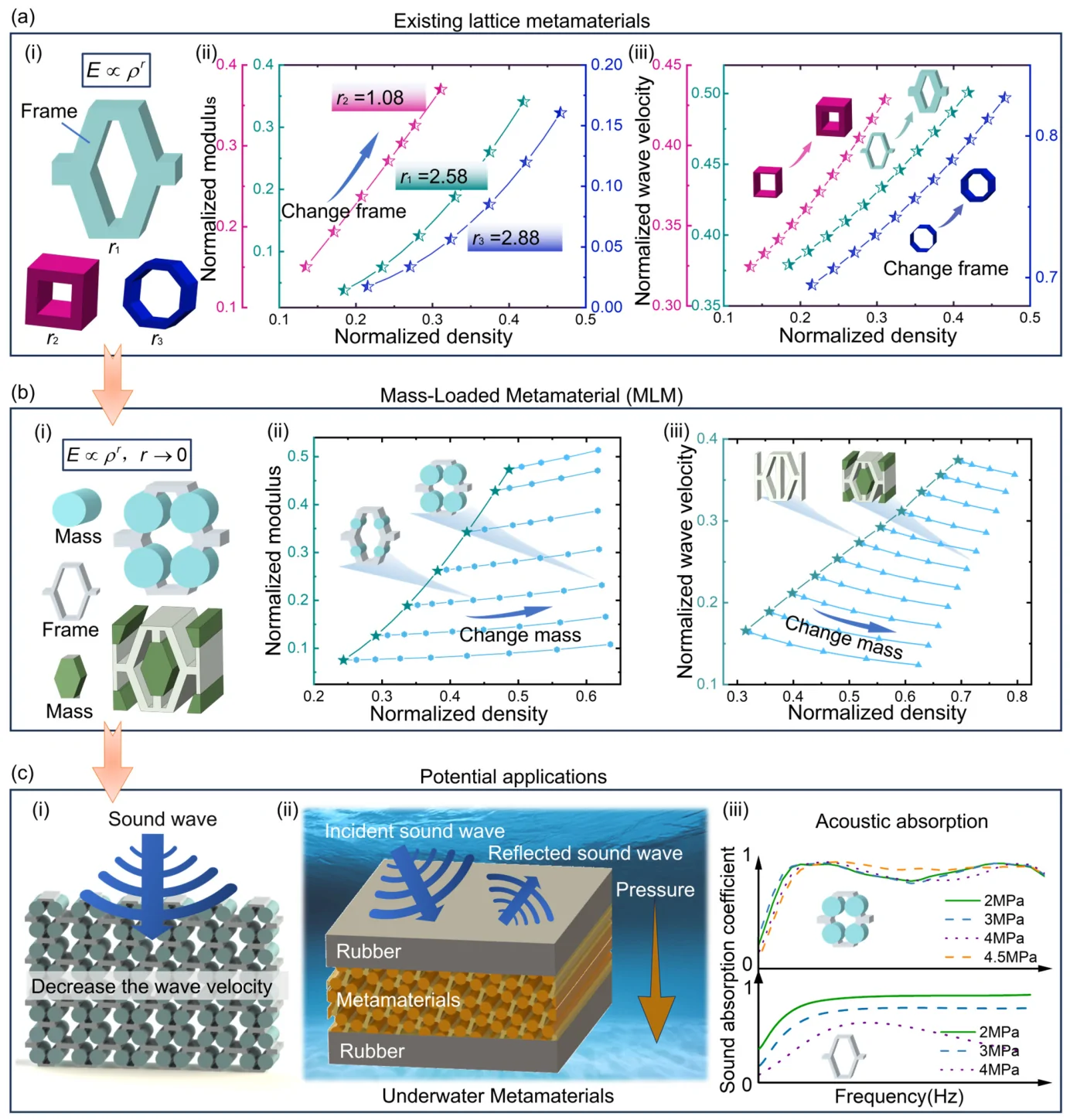
(**a**) Schematics and properties of existing lattice materials: (**i**) structural configurations; (**ii**) power-law scaling relationship between modulus and relative density; (**iii**) variation in wave velocity with relative density. (**b**) Schematic illustration and property characterization of MLM: (**i**) structural configuration; (**ii**) modulus–density decoupling realized by tailoring circular mass block dimensions; (**iii**) wave velocity–density decoupling achieved by adjusting hexagonal mass block sizes. (**c**) Application prospects of MLM: (**i**) schematic of acoustic wave modulation; (**ii**) underwater sound absorption structure with hydrostatic pressure increasing with depth and MLM embedded in rubber matrices; (**iii**) comparative diagram showing the sound absorption advantages of MLM in pressure-resistant, low-frequency, and broadband scenarios.

The dominant design paradigm of elastic metamaterials is that of single-phase lattice metamaterials, which consist of solid constituents and internal voids. As shown in Figure 1a(i,ii), lattice metamaterials typically exhibit a power-law correlation between their Young’s modulus and density [28,29,30], i.e., $E\propto \rho^{r}$, where *E* is the modulus and *ρ* is the density. The exponent *r* depends on the specific material distribution and the dominant deformation mechanism, ranging from one to three [31,32]. This well-established power-law relationship prevents the decoupling between static mechanical properties and dynamic wave propagation behaviors [33,34,35].

To further understand the dynamic implications of this coupling, it is essential to examine wave velocity and acoustic impedance, two key quantities that link static properties to wave propagation behavior [36,37]. In solid media, sound propagates in the form of elastic waves, whose velocity is governed by the static modulus and density, denoted by $v\propto \sqrt{E/\rho }$. Acoustic impedance is defined as the product of density and wave velocity, namely Z=ρv. Thus $Z=\sqrt{E\rho }$ is obtained, indicating that the wave-related properties are intrinsically linked to the static modulus and density. As illustrated in Figure 1a(iii), the modulus increases exponentially with density, leading to a synchronous increase in wave velocity [38,39]. Acoustic impedance is similarly constrained by this coupling effect, limiting its independent tunability. An intriguing question thus arises: how can different relationships among acoustic impedance (*Z*), modulus (*E*), and wave velocity (*v*) be realized to break through such limitations?

Motivated by this question, this work targets the regime where the power-law exponent *r* approaches zero, so that high modulus and high acoustic impedance can coexist with low wave velocity [40]. Pentamode metamaterials showed partial decoupling of modulus and density [41,42], but their slender connections offer little stiffness under hydrostatic pressure. The proposed design, referred to as mass-loaded metamaterial (MLM), takes a different approach. It uses a hexagonal framework as the load-bearing skeleton, with mass blocks placed in low-stress voids away from the main force paths (Figure 1b(i). Under pressure, the framework remains intact, while the blocks add density without stiffening the structure. The fitted power-law exponent drops toward zero (Figure 1b(ii)), and the wave velocity falls as the modulus rises (Figure 1b(iii)).

The MLM differs fundamentally from existing designs. In conventional mass-loaded structures, the modulus and density are inherently coupled, such that an increase in modulus is inevitably accompanied by a rise in density [1,34]. Locally resonant metamaterials rely on resonant mechanisms, which inherently suffer from narrow bandwidths and make it difficult to simultaneously achieve high stiffness and favorable dynamic performance [43]. Pentamode materials sacrifice stiffness to lower the wave velocity and exhibit poor mechanical stability under hydrostatic pressure [41,44]. In topology-optimized structures, decoupling often emerges merely as an incidental byproduct of optimization rather than as a deliberately engineered objective [45]. In the MLM, by contrast, mass blocks are deliberately positioned in low-stress regions away from the primary load-bearing paths, so that the density increases while the framework stiffness remains nearly unchanged. This static decoupling further extends into the dynamic regime: by synergistically tuning the wall thickness and the mass block size of the MLM, the modulus can be increased while the wave velocity decreases (Figure 1c(i)) and the acoustic impedance rises. This combination of properties, which is mutually exclusive in conventional materials, offers a new route to achieving broadband sound absorption under high hydrostatic pressure [46,47,48,49,50,51] while reconciling acoustic performance with structural integrity (Figure 1c(ii,iii)).

A quantitative comparison of the proposed MLM with recently reported pressure-resistant underwater absorbers is provided in Supplementary Material S1.

## 2. Structural Design, Decoupling Characteristics, and Experimental Verification of MLM

### 2.1. Structural Design of MLM

**Figure 2:**
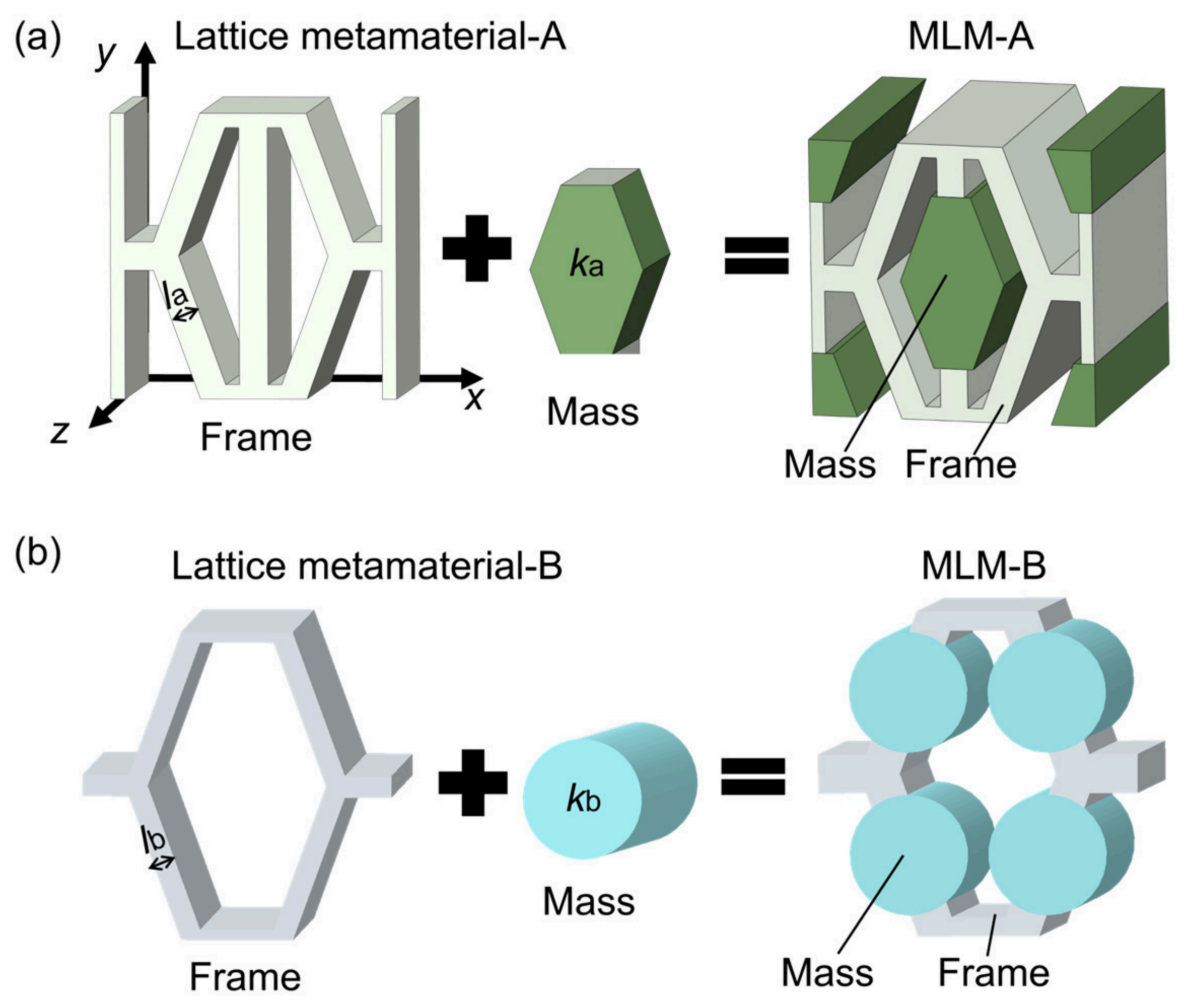
Design of MLM. (**a**) Assembly process of MLM-A: a hexagonal mass block (controlled by parameter *k*_a_) is integrated into the central pillar of lattice metamaterial-A with wall thickness *l*_a_. (**b**) Assembly process of MLM-B: circular mass blocks (controlled by parameter *k*_b_) are loaded onto the lateral pillars of lattice metamaterial-B with wall thickness *l*_b_.

MLM is built by adding mass blocks to the frame of existing lattice materials, with two configurations shown in Figure 2. In MLM-A, hexagonal mass blocks are placed in the central void of a hexagonal lattice with wall thickness *l*_a_; their size is set by the dimensionless ratio *k*_a_ (Figure 2a). In MLM-B, circular mass blocks are placed on the four lateral pillars of a hexagonal lattice with wall thickness *l*_b_; their radius is set by *k*_b_ (Figure 2b). Both unit cells measure 10 mm × 10 mm × 10 mm, with the *z*-direction used for uniaxial tension.

The ranges of the four design variables were determined by manufacturing constraints and geometric compatibility. The lower bounds of *l*_a_ (0.7 mm) and *l*_b_ (0.5 mm) correspond to the minimum reliably printable wall thickness for PEEK in the selected additive manufacturing process; below these values, wall defects and poor interlayer adhesion become significant. The upper bounds (*l*_a_ = 1.7 mm, *l*_b_ = 1.2 mm) are set by the condition that the mass blocks must not contact the surrounding walls at the maximum mass-loading ratio. Beyond these limits, the internal voids become too small to accommodate the blocks, and the structure degenerates toward a solid medium. The mass-loading ratio range *k* = 0.1–0.8 was chosen to span the transition from weak to strong loading. Below *k* = 0.1, the blocks are too small to produce a meaningful density change; above *k* = 0.8, they approach the load-bearing walls and risk being drawn into the load path, which would reintroduce modulus–density coupling. Within these bounds, stress analysis confirms that the blocks remain in low-stress regions under uniaxial loading. Detailed geometric definitions are given in Supplementary Material S2.

### 2.2. Decoupling of Modulus and Density in MLM

**Figure 3:**
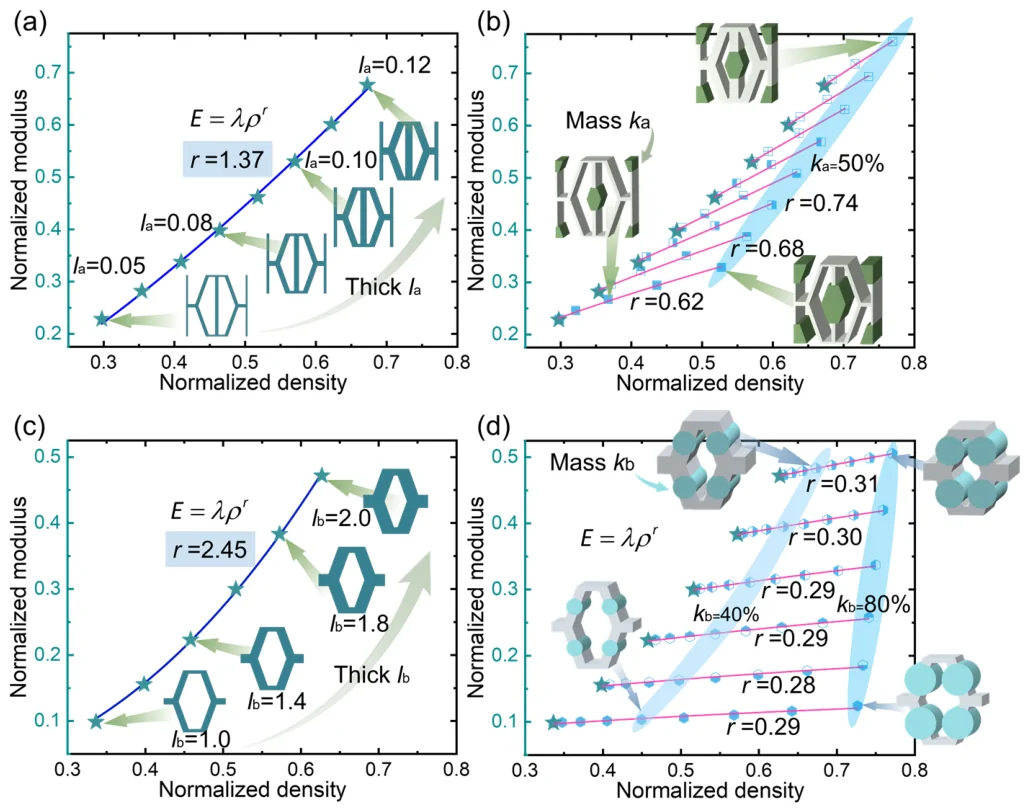
Scatter plots and fitting curves of normalized modulus and normalized density under different wall thicknesses and mass block ratios. (**a**) For lattice metamaterial-A varying with wall thickness *l*_a_, modulus and density show a strong correlation, with the fitting coefficient *r* = 1.37. (**b**) Variation trends of modulus and corresponding fitting curves of MLM-A when gradually increasing the occupancy ratio *k*_a_ of the central hexagonal mass block under different wall thicknesses, where the fitting coefficient r < 1, where the shadow area represents the same mass block occupancy ratio (the same below). (**c**) For lattice metamaterial-B varying with wall thickness lb, modulus and density show a strong correlation, with the fitting coefficient *r* = 2.45. (**d**) Variation trends of modulus and corresponding fitting curves of MLM-B when gradually increasing the occupancy ratio *k*_b_ of the side mass block under different wall thicknesses, where the fitting coefficient approaches zero.

The modulus–density relationship was obtained by independently varying *l* and *k*. The numerical method is depicted in Supplementary Material S3. Since *E_x_* and *E_y_* exhibit similar trends, only *E_y_* is shown in Figure 3; *E_x_* is given in Appendix A.

Figure 3a shows the modulus of lattice metamaterial-A versus density as *l*_a_ changes; Figure 3c gives the corresponding results for lattice metamaterial-B with varying *l*_b_. Figure 3b,d add the MLM-A and MLM-B curves, obtained by increasing *k*_a_ and *k*_b_ at each fixed wall thickness. Mass blocks clearly slow the growth of modulus with density: the modulus can be adjusted at fixed density, or vice versa.

For quantitative verification, the data were fitted with $E=\lambda \rho^{r}$. As listed in Table 1, all R^2^ values exceed 0.9, and the fitting error and 95% confidence intervals of r are provided.

Fitting the data to $E=\lambda \rho^{r}$ gives *r* = 1.37 for lattice metamaterial-A and *r* = 2.45 for lattice metamaterial-B. At fixed wall thickness, adding mass blocks lowers *r* to 0.620 for MLM-A and 0.287 for MLM-B. For MLM-B, the exponent *r* decreases by approximately 88%. As can be directly observed in Figure 3d, the upward trend of modulus with density is significantly slowed, shifting the modulus–density mapping from a one-to-one correspondence to a many-to-many relationship: the same modulus can be achieved at different densities, and the same density can yield different moduli.

The mass blocks are located in low-stiffness regions, so they increase the density without altering the load-bearing paths or the stiffness distribution. However, the blocks are not perfectly isolated: each block connects to the framework along its boundary, and this connection contributes a small amount of stiffness. Consequently, the positive correlation between modulus and density is far weaker than in conventional materials, which is the origin of the observed decoupling effect.

### 2.3. Experimental Verification of MLM Decoupling Characteristics

**Figure 4:**
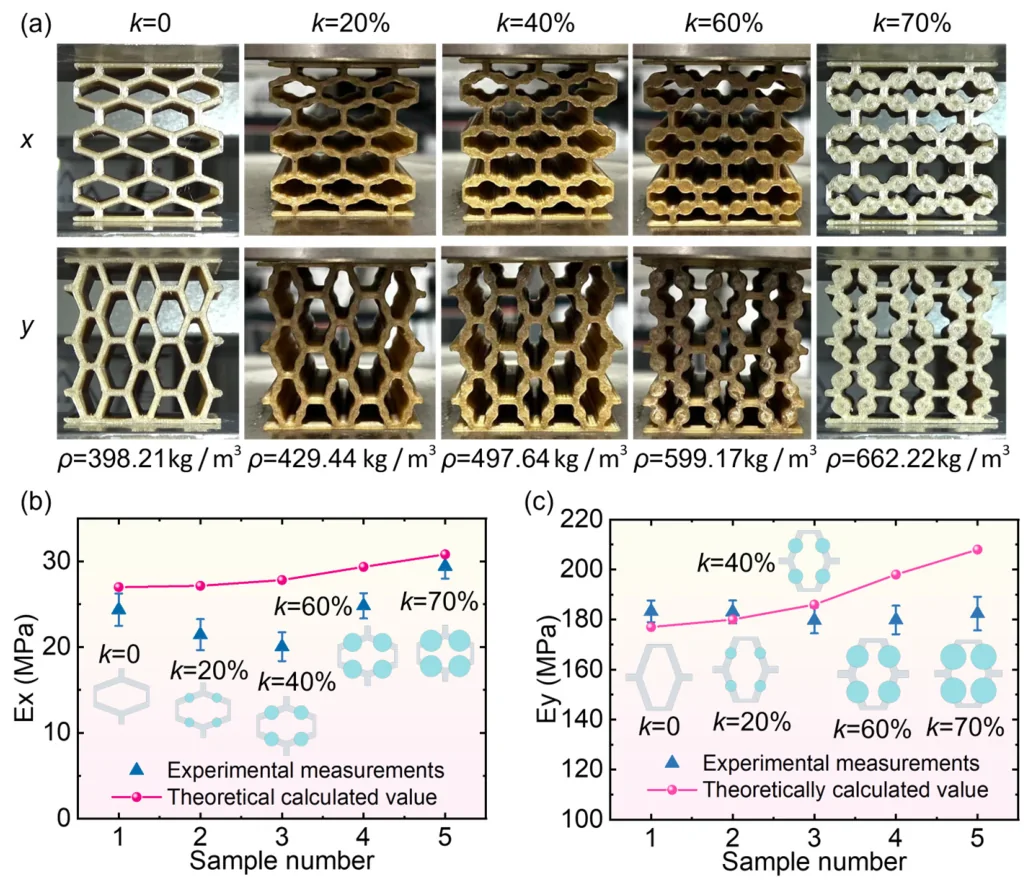
Photographs of 3D-printed MLM array specimens and the measured Young’s modulus. (**a**) Compression test specimens fabricated from PEEK with varying mass block ratios (*k* = 0, 20%, 40%, 60%, and 70%) for both *x*- and *y*-direction loading, along with their corresponding densities. (**b**) Young’s modulus for the MLM along the *x*-direction. (**c**) Young’s modulus for the MLM along the *y*-direction.

To verify the decoupling characteristics, five groups of MLM-B array specimens were fabricated with the same wall thickness but different mass block ratios (*k* = 0, 20%, 40%, 60%, 70%). Both the mass blocks and the hexagonal framework were 3D-printed from polyether ether ketone (PEEK), and the material properties of PEEK are listed in Table 2, with the printing parameters detailed in Supplementary Material S4. The MLM-B configuration was selected because numerical simulations indicated that it exhibits a more pronounced decoupling effect. Each specimen consisted of a 3 × 3 array of unit cells. Compression tests were conducted along the *x*- and *y*-directions (Figure 4a). As the mass block ratio *k* increased from 0 to 70%, the equivalent density increased from 383 to 637 kg/m^3^. One specimen was tested for each parameter condition, subjected to four repeated loading–unloading cycles with a maximum strain of 1%. The elastic moduli in the *x*- and *y*-directions were obtained by linear fitting over the 0.5–1% strain segment of each cycle, as shown in Figure 4b,c. The error bars represent the standard deviation of four repeated measurements on the same specimen, reflecting the repeatability of the measurement system. The compression tests were performed on an electronic universal testing machine (INSTRON, Norwood, MA, USA), model 5982.

In Figure 4b,c, the measured *x*-direction modulus ranges from 20 to 30 MPa, while the *y*-direction modulus remains stable near 180–185 MPa; the circular dotted lines represent the numerical simulation results. As the specimen density increases (a 66% increase), no significant rise in modulus is observed in either direction, indicating a decoupling trend in the experimental results. The numerical model with *r* = 0.287 predicts an approximately 16% increase in modulus over this density range; however, the measured modulus increase is clearly smaller than this prediction, suggesting that the actual decoupling effect is more pronounced than the numerical prediction. This discrepancy is attributed to two main factors. First, the difference in boundary conditions: the simulation employs periodic boundary conditions to represent an infinite structure, whereas the experimental specimens are finite-sized unit cell arrays. Second, certain errors exist in the specimen printing and measurement processes. Therefore, the residual coupling strength in the actual structure is likely lower than the value predicted by *r* = 0.287.

## 3. Wave Regulation via MLM

### 3.1. Anomalous Modulation of Acoustic Velocity

In a solid, wave velocity depends on both modulus and density $v\propto \sqrt{E/\rho }$. Lowering the modulus reduces the velocity [52,53], but in conventional materials density and modulus rise together, so velocity cannot be lowered by adding density alone. The MLM removes this constraint. Because its modulus stays nearly constant as density increases, the velocity drops with density, producing the inverse modulus–velocity relation shown below.

Consider the two-dimensional MLM. The displacement field is denoted by $u={\left[u_{1},u_{2}\right]}^{T}$, where $u_{i}=u_{i}(x_{1},x_{2},t)$ for i=1,2. The equation of motion is given by

(1) $$\rho \frac{\partial^{2}u_{i}}{\partial t^{2}}=\frac{\partial \sigma_{ij}}{\partial x_{j}},\;i,j=1,2,$$

where $x_{j}$ denotes the spatial coordinate, *t* denotes the time, and $\sigma_{ij}$ denotes the Cauchy stress tensor component. By substituting the constitutive relationship $\sigma_{ij}=C_{ijkl}\varepsilon_{kl}$, $\varepsilon_{kl}=\frac{1}{2}\left(\frac{\partial u_{k}}{\partial x_{l}}+\frac{\partial u_{l}}{\partial x_{k}}\right)$ and strain into Equation (1), $C_{ijkl}$ denotes the elastic stiffness tensor, and by utilizing its symmetry for simplification, we obtain

(2) $$\rho \frac{\partial^{2}u_{i}}{\partial t^{2}}=C_{ijkl}\frac{\partial^{2}u_{k}}{\partial x_{j}\partial x_{l}}.$$

Assuming a plane wave propagates in the MLM along the unit direction vector $n=(n_{1},n_{2})$, where ${n_{1}}^{2}+{n_{2}}^{2}=1$, the displacement field can be expressed as:

(3) $$u_{k}=A_{k}e^{i(\omega t-K(n_{1}x_{1}+n_{2}x_{2}))},k=1,2.$$

where ω is the angular frequency, K=ω/v is the wavenumber, *v* is the phase velocity, and $A_{k}$ is the complex amplitude (polarization vector). The direction components are $n_{1}=\cos\theta ,n_{2}=\sin\theta$, θ represents the angle between the incident acoustic wave and the normal vector of the interface. Substituting (3) into (2) and noting that $\omega^{2}=K^{2}v^{2}$ we obtain

(4) $$\rho v^{2}A_{i}=C_{ijkl}n_{j}n_{l}A_{k}.$$

Define the Christoffel matrix Γ(n) (which does not include density) as:

(5) $$\Gamma_{ik}=C_{ijkl}n_{j}n_{l},\;i,k=1,2.$$

Equation (4) then becomes:

(6) $$\left(\Gamma_{ik}-\rho v^{2}\delta_{ik}\right)A_{k}=0,$$

where $\delta_{ik}$ is the Kronecker delta, and $\Gamma_{ik}$ is the two-dimensional Christoffel matrix. In the following, $C_{ijkl}$ is denoted by the Voigt notation C, as follows:

(7) $$C=\left(\begin{array}{ccc} C_{11} & C_{12} & C_{16}\\ C_{12} & C_{22} & C_{26}\\ C_{16} & C_{26} & C_{66} \end{array}\right),$$

then, the Equation (5) is rewritten as

(8) $$\left\{\begin{array}{c} \;\Gamma_{11}=C_{11}n_{1}^{2}+C_{66}n_{2}^{2}+2C_{16}n_{1}n_{2}\\ \;\Gamma_{22}=C_{66}n_{1}^{2}+C_{22}n_{2}^{2}+2C_{26}n_{1}n_{2}\\ \Gamma_{12}=\Gamma_{21}=C_{16}n_{1}^{2}+C_{26}n_{2}^{2}+(C_{12}+C_{66})n_{1}n_{2} \end{array}\right..$$

The characteristic equation is simplified to

(9) $$\det\left(\begin{array}{cc} \Gamma_{11}-\rho v^{2} & \Gamma_{12}\\ \Gamma_{12} & \Gamma_{22}-\rho v^{2} \end{array}\right)=0.$$

The characteristic solutions to Equation (9) are as follows:

(10) $$\begin{array}{l} V_{L}(\theta )=\sqrt{\frac{\Gamma_{11}+\Gamma_{22}+\sqrt{{\left(\Gamma_{11}-\Gamma_{22}\right)}^{2}+4\Gamma_{12}^{2}}}{2\rho }},\\ V_{S}(\theta )=\sqrt{\frac{\Gamma_{11}+\Gamma_{22}-\sqrt{{\left(\Gamma_{11}-\Gamma_{22}\right)}^{2}+4\Gamma_{12}^{2}}}{2\rho }}. \end{array}$$

Considering the condition of normal incidence (θ=0°), the characteristic solutions are expressed as:

(11) $$\begin{array}{c} V_{L}=\sqrt{\frac{C_{11}+C_{66}+\sqrt{{\left(C_{11}-C_{66}\right)}^{2}+4C_{16}^{2}}}{2\rho },}\\ V_{S}=\sqrt{\frac{C_{11}+C_{66}-\sqrt{{\left(C_{11}-C_{66}\right)}^{2}+4C_{16}^{2}}}{2\rho }.} \end{array}$$

In Equation (11), the faster velocity *V*_L_ corresponds to the longitudinal wave, whereas the slower velocity *V*_S_ denotes the shear wave [54,55,56]. At a normal incidence angle (i.e., incident angle θ=0°), the propagation velocities of both longitudinal and shear waves exhibit a strong correlation with the stiffness matrix C and material density. Beyond the decoupling between modulus, the MLM also achieves the decoupling between the density and elastic constants *C*_ij_, which is provided in Appendix B. This unique decoupling enables the reduction in acoustic wave velocity through independent density modulation, leading to the counterintuitive phenomenon wherein acoustic velocity decreases with increasing modulus.

**Figure 5:**
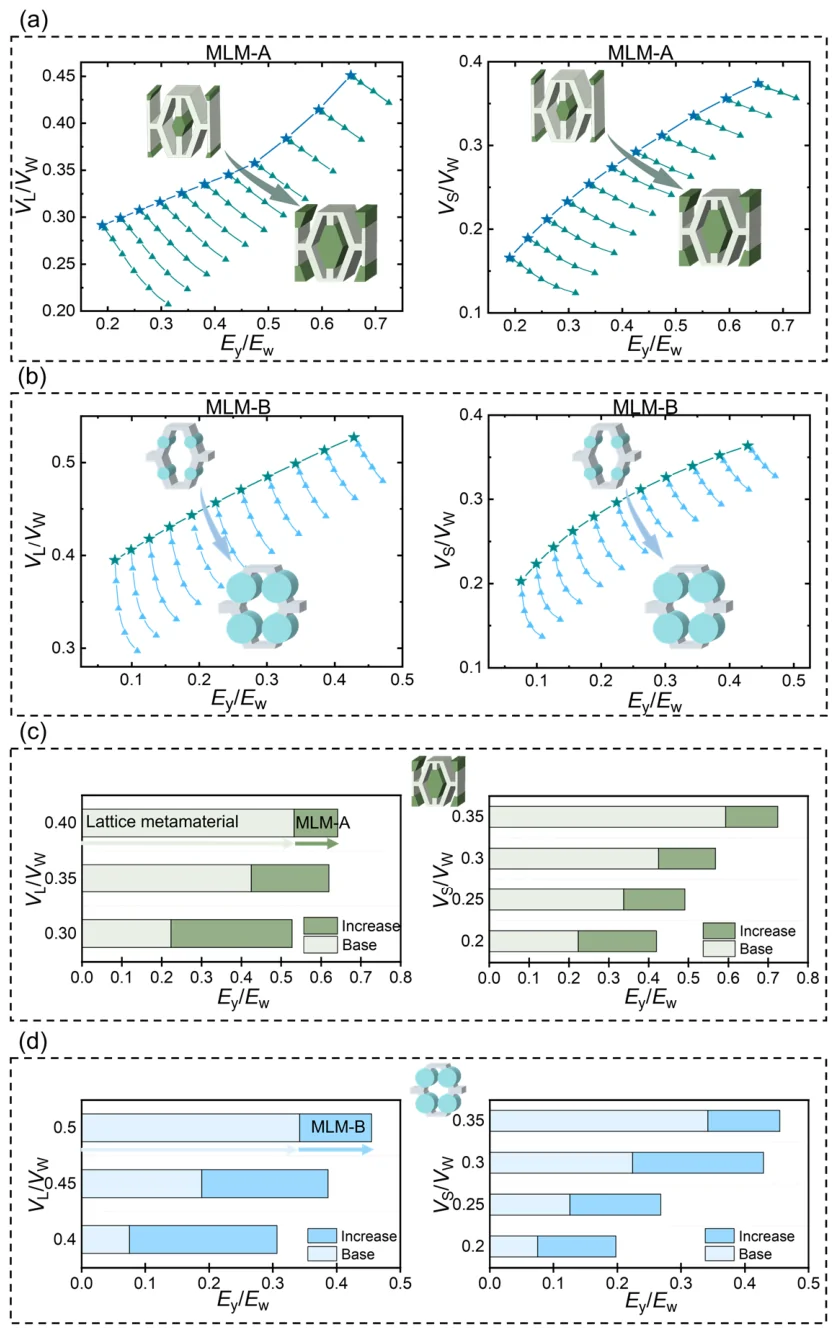
Variations in longitudinal and shear wave velocities of MLM-A and MLM-B with modulus. (**a**) Normalized longitudinal wave velocity (*V*_L_/*V*_w_) and shear wave velocity (*V*_S_/*V*_w_) as functions of normalized modulus (*E*_y_/*E*_w_) for MLM-A. (**b**) Normalized longitudinal wave velocity (*V*_L_/*V*_w_) and shear wave velocity (*V*_S_/*V*_w_) as functions of normalized modulus (*E*_y_/*E*_w_) for MLM-B. (**c**) Bar charts of modulus enhancement under constant acoustic wave velocity. The light green bar denotes the normalized modulus *E*_y_/*E*_w_ of the unloaded structure, while the green shaded region represents the increment range of *E*_y_/*E*_w_ for MLM-A relative to the baseline. (**d**) Bar charts of modulus enhancement under constant acoustic wave velocity. The light blue bar denotes the normalized modulus *E*_y_/*E*_w_ of the unloaded structure, while the blue shaded region represents the increment range of *E*_y_/*E*_w_ for MLM-B relative to the baseline.

Under normal incidence, the longitudinal and shear wave velocities are jointly determined by the stiffness matrix and density. By synergistically tuning the wall thickness *l* and the mass block ratio *k* of the MLM, the acoustic velocity of MLM-A can be reduced by 10–30% and that of MLM-B by 7–35%, while the modulus remains nearly unchanged, as shown in Figure 5a,b. Figure 5c,d demonstrate that the modulus can be enhanced by 20–307% while the acoustic velocity remains nearly constant.

### 3.2. Anomalous Modulation of Acoustic Impedance

**Figure 6:**
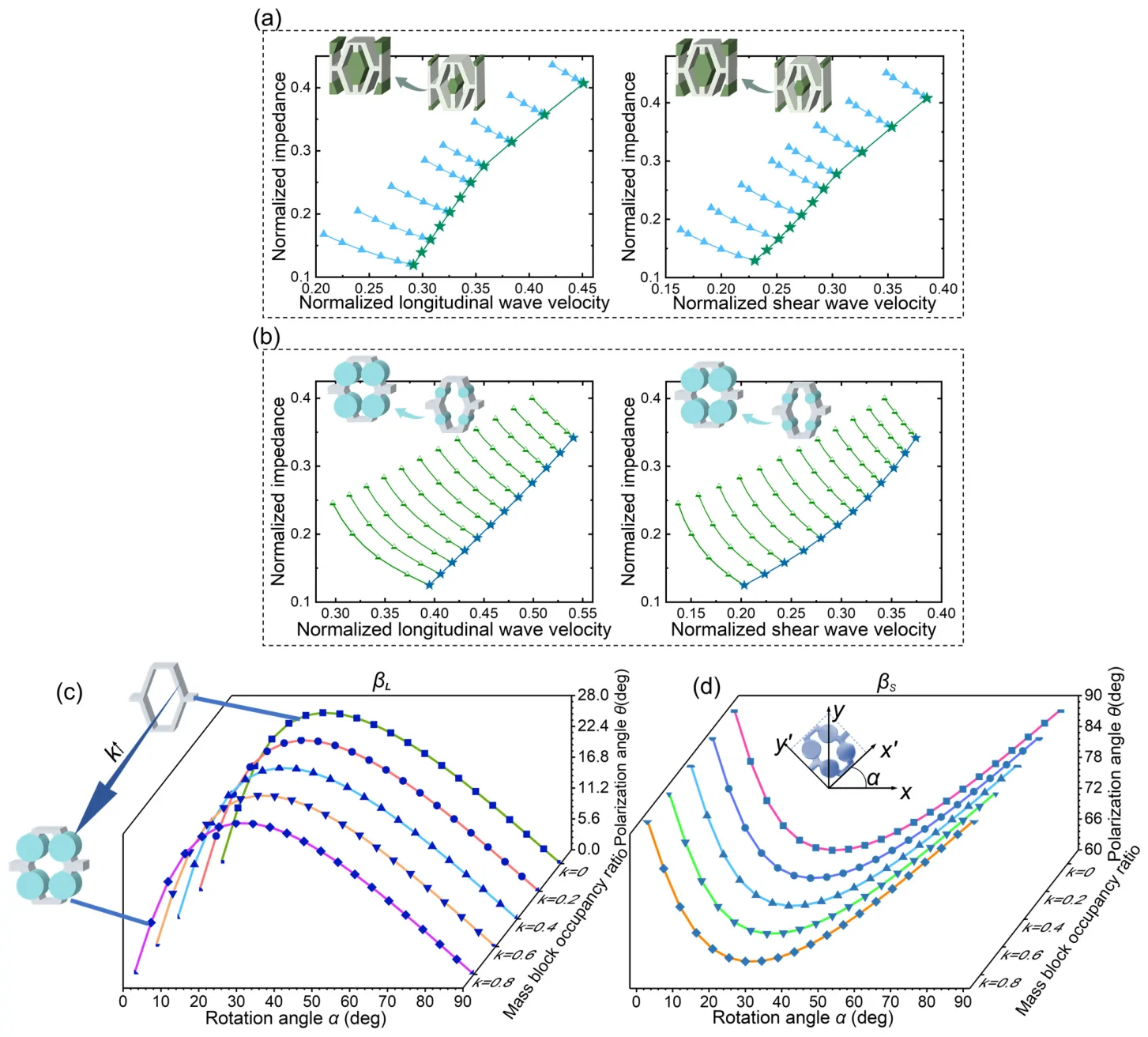
Acoustic impedance and polarization angle modulation of MLM. (**a**) Normalized acoustic impedance as a function of normalized longitudinal and shear wave velocities for MLM-A. Star-marked curves: impedance variation via wall thickness *l*; triangle-marked curves: via mass block ratio *k* at fixed *l*. Insets show structural evolution at different wave velocity levels. (**b**) Corresponding results for MLM-B. (**c**) Polarization angle of longitudinal waves versus structural rotation angle for *k* = 0, 0.2, 0.4, 0.6, 0.8. The angle increases from 0° to a maximum and then decreases, enabling longitudinal–shear wave conversion. Insets show structures at different *k*. (**d**) Polarization angle of shear waves versus rotation angle for the same *k* values. The angle starts at 90°, decreases and then rises, symmetric to the longitudinal case.

Acoustic impedance, defined as Z=ρv, is jointly determined by the material’s density (ρ) and acoustic velocity (v). This constitutive relation implies that, in conventional material systems, achieving high impedance inherently requires the simultaneous attainment of high wave velocity and high density [57]. Consequently, in existing lattice metamaterials, acoustic impedance varies synchronously with acoustic velocity. As evidenced by the star-shaped curves in Figure 6a,b, varying the wall thickness of lattice-A and lattice-B yields a positive correlation between acoustic impedance and both longitudinal and shear wave velocities.

The proposed MLM design transcends the inherent limitations of existing lattice metamaterials, achieving an anomalous acoustic characteristic: acoustic impedance increases with decreasing wave velocity. Modulating the mass fraction of the MLM at a fixed wall thickness fundamentally inverts this relationship, yielding an inverse correlation between impedance and wave velocity. For both MLM-A and MLM-B, the increase in acoustic impedance is accompanied by a decrease in both longitudinal and shear wave velocities, enabling the simultaneous achievement of high impedance and low wave velocity.

### 3.3. Polarization Angle Modulation

The polarization angle β, defined as the angle between the wave propagation direction and the vibration direction, fundamentally governs the propagation characteristics of acoustic waves [58,59,60]. In the limiting cases, a longitudinal wave with β=0° corresponds to a pure compressional mode with vibration parallel to propagation, while a shear wave with β=90° represents a pure shear mode characterized by vibration perpendicular to propagation [60,61,62,63]. Notably, the polarization angle can be obtained from the wave dynamics equations, the displacement–stress relationship, and the stiffness matrix in Equation (7); the resulting polarization angles for shear and longitudinal waves are presented in Equation (12):

(12) $$\begin{array}{l} \beta_{L}=\tan^{-1}\frac{2C_{16}}{C_{11}-C_{66}+\sqrt{{\left(C_{11}-C_{66}\right)}^{2}+4{\left(C_{16}\right)}^{2}}}\\ \beta_{S}=\tan^{-1}\frac{2C_{16}}{C_{11}-C_{66}-\sqrt{{\left(C_{11}-C_{66}\right)}^{2}+4{\left(C_{16}\right)}^{2}}}. \end{array}$$

When the polarization angle deviates from these two special cases (0° < β < 90°), the propagating waves are neither purely longitudinal nor purely shear, but instead exhibit coupled deformation behavior. Under such conditions, acoustic excitation induces simultaneous deformation in both the longitudinal and transverse directions, leading to coupling between compressional and shear deformations. This coupled response provides the physical underpinning for mode conversion between longitudinal and shear waves, a mechanism that enables incident longitudinal waves to partially convert into shear waves.

In the proposed MLM design, the polarization angle can be effectively tuned by varying the orientation angle *α* of the unit cell. Notably, this modulation depends solely on the geometric orientation and remains independent of the mass block parameters. As shown in Figure 6c, under different mass block ratios (*k*), both the magnitude of the polarization angle and its trend with respect to the orientation angle remain consistent. This characteristic provides a robust and decoupled control strategy for achieving wave mode conversion.

### 3.4. Experimental Verification of Acoustic Velocity Reduction

**Figure 7:**
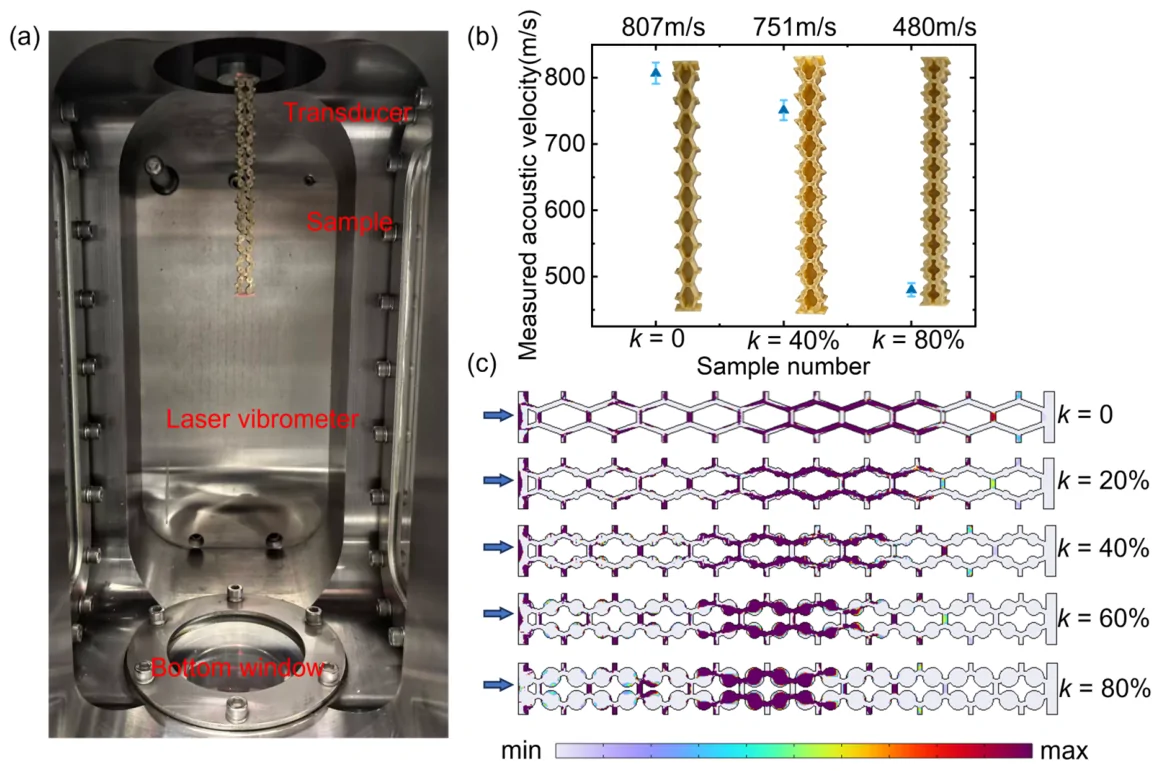
Experimental verification of acoustic velocity modulation for MLM samples. (**a**) Schematic diagram of the broadband resonance test setup for acoustic velocity measurement, consisting of an acoustic transducer, test specimen, laser vibrometer and optical bottom window. (**b**) 3D-printed PEEK specimens with mass-loading ratios *k* = 0, 40%, and 80%; measured acoustic velocities (at room temperature, 26 °C, under ambient pressure). (**c**) Transient wave propagation contours obtained from time-domain numerical simulations for MLMs with various mass-loading ratios *k* = 0, 20%, 40%, 60% and 80%, where the color bar represents the normalized vibration amplitude from minimum to maximum.

This study follows the method of Liu et al. [64] to measure the acoustic velocity by direct wave extraction, with cross-validation using the resonance method. The specimen was fixed at the bottom of an impedance tube and excited by a short broadband Butterworth pulse. A laser vibrometer recorded the signals at both ends, and the acoustic velocity was obtained by direct wave extraction combined with resonance peak analysis (Figure 7a).

Three groups of specimens (*k* = 0, 40%, 80%) were fabricated from PEEK, each group containing two parallel specimens with dimensions of 10 mm × 10 mm × 100 mm (Figure 7b). The measured acoustic velocities at *k* = 0, 40%, and 80% were 807, 751, and 480 m/s, respectively. The measurement uncertainty is approximately 10%, arising from dimensional tolerances (~2%), temperature fluctuations (~4%), and signal processing (~4%); the root-sum-square combined uncertainty is about 6%, so 10% is a conservative estimate. Since only two specimens were included in each group, the data provide trend-level validation. The monotonic decreasing trend of the acoustic velocity with *k* is consistent with the calculated results. Moreover, the acoustic velocity in the time-domain simulations shown in Figure 7c exhibits the same monotonic decreasing trend, further supporting the velocity-reduction capability of the MLM.

The increase in modulus combined with a limited increase in density leads to a reduction in acoustic velocity. The acoustic impedance Z=ρv, calculated from the measurement results is 3.2 × 10^5^, 3.7 × 10^5^, and 3.8 × 10^5^ kg/(m^2^·s), increasing with *k*. This combination of rising modulus, decreasing velocity, and rising impedance constitutes the physical basis of this work.

### 3.5. Design Guidelines for Independent Tailoring of Modulus and Acoustic Velocity

Based on the numerical homogenization and experimental results above, a simple design rule emerges. The wall thickness *l* controls the modulus *E*: a larger *l* gives a higher *E* (Figure 3a,c). The mass-loading ratio *k* controls the density *ρ*, and hence the velocity *v*. Because the mass blocks sit in low-stiffness regions, increasing *k* raises *ρ* without much change in the load-bearing paths or stiffness distribution (Figure 3b,d).

To reach a target modulus, choose *l* from the *E*–*l* relation. Once *l* is fixed, the modulus stays nearly constant over a wide range of *k* (Figure 4b,c). To reach a target velocity, adjust *k* at the chosen *l*. As *k* increases, *v* drops while *E* remains nearly unchanged (Figure 5a,b and Figure 7b). The two parameters can thus be tuned separately: *l* for modulus, *k* for velocity. They can of course also be tuned together when a combined adjustment of both properties is needed.

## 4. Sound Absorption Application

### 4.1. Sound Absorption Mechanism of MLM

**Figure 8:**
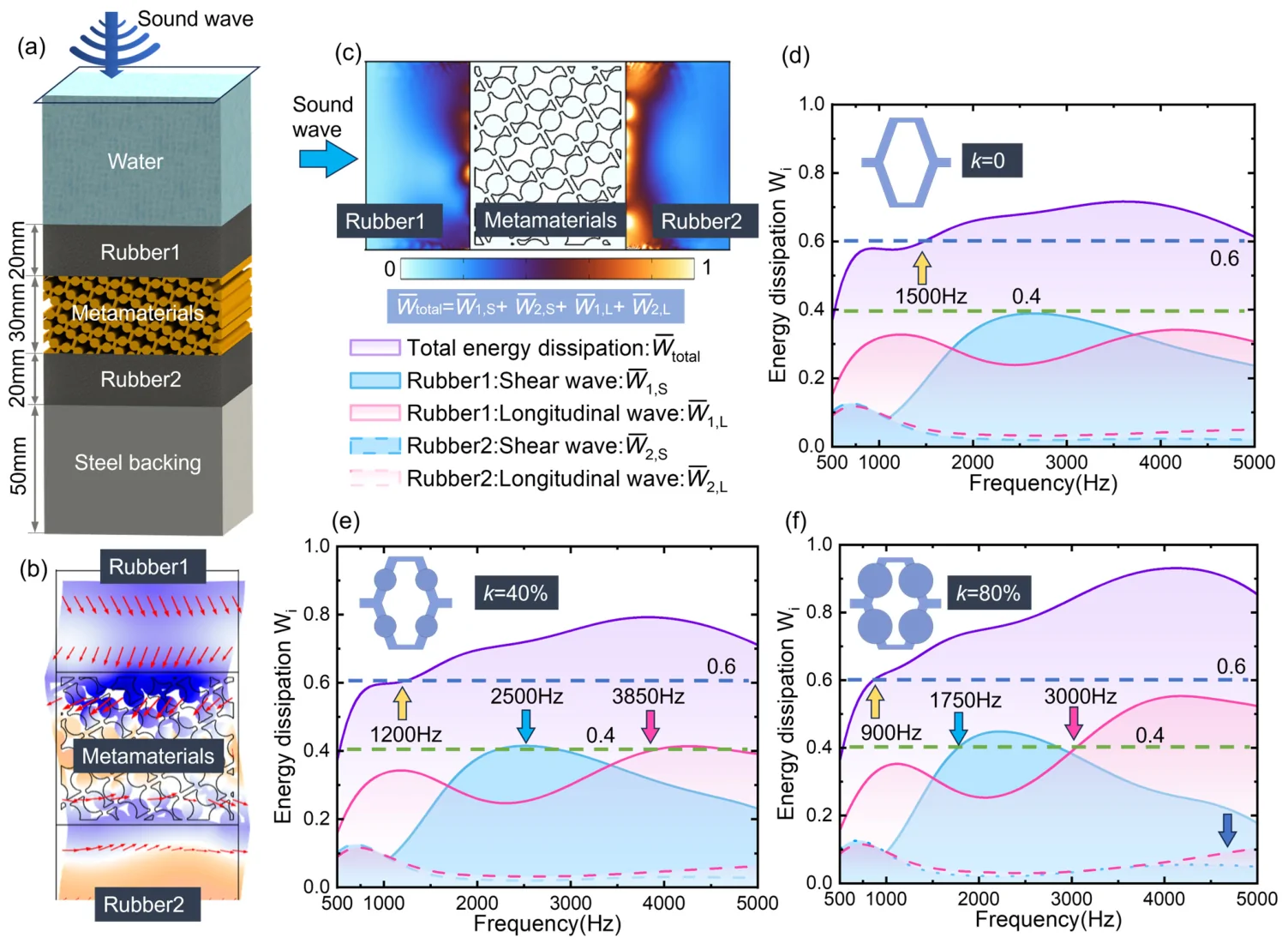
Sound absorption mechanism and energy dissipation analysis of the MLM. (**a**) Underwater sound absorption simulation model: the MLM is sandwiched between two rubber layers on a steel backing, with normally incident plane waves from the water domain and periodic boundary conditions applied on the left and right sides. (**b**) Schematic of the structural displacement after acoustic incidence: after being modulated by the MLM, the predominantly vertical displacement in rubber1 is converted into shear-dominated displacement in rubber2. (**c**) Spatial distribution of rubber1 and rubber2, with the acoustic wave incident perpendicular to the surface from the rubber1 side. (**d**–**f**) Energy dissipation spectra of MLM-B at *k* = 0, 40%, and 80%: the purple solid line represents the total dissipation ${\bar{W}}_{\mathrm{total}}$; the blue solid/dashed lines represent the shear/longitudinal dissipation ${\bar{W}}_{1,S}$/${\bar{W}}_{1,L}$ in rubber1; the pink solid/dashed lines represent the shear/longitudinal dissipation ${\bar{W}}_{2,S}$/${\bar{W}}_{2,L}$ in rubber2.

Rubber and similar viscoelastic materials can dissipate acoustic energy, and embedding acoustic structures into a rubber matrix is a common approach in sound absorption [65,66]. Following this strategy, the MLM is combined with rubber to form an absorbing composite in this work. The absorption coefficient is calculated by the finite element method. The model is shown in Figure 8a: the MLM is sandwiched between rubber layers on a steel backing, normally incident plane waves impinge from the water domain, and periodic boundary conditions are applied on the left and right sides to represent an infinite array. Detailed modeling parameters are provided in Supplementary Material S5.

Figure 8b presents a schematic of the structural displacement after acoustic wave incidence. After the normally incident wave passes through the metamaterial, the predominantly vertical displacement in rubber1 is converted into shear-dominated displacement in rubber2. As can be seen from the curves in Figure 8c–f, shear deformation within the rubber layers is the dominant dissipation mechanism, and the energy dissipation caused by longitudinal deformation remains below 15% across the broadband frequency range.

To further quantitatively analyze the advantages of the MLM over the lattice metamaterial in terms of the sound absorption mechanism, Figure 8c indicates the spatial distribution of rubber1 and rubber2, with the acoustic wave incident perpendicular to the surface from the rubber1 side. The legend for Figure 8d–f is as follows: the purple solid line represents the total energy dissipation ${\bar{W}}_{\mathrm{total}}$, which lies at the top in all three panels; the blue solid and dashed lines represent the shear-wave energy dissipation ${\bar{W}}_{1,S}$ and longitudinal-wave energy dissipation ${\bar{W}}_{1,L}$ in rubber1, respectively; the pink solid and dashed lines represent the shear-wave energy dissipation ${\bar{W}}_{2,S}$ and longitudinal-wave energy dissipation ${\bar{W}}_{2,L}$ in rubber2, respectively. All are normalized energy dissipation values, and the calculation method is provided in Supplementary Material S6.

Figure 8d, Figure 8e, and Figure 8f show the energy dissipation of MLM-B at mass block ratios of *k* = 0, 40%, and 80%, respectively. As *k* increases, the total energy dissipation curve rises continuously across the entire frequency range. At *k* = 80%, the bandwidth over which the total energy dissipation exceeds 0.6 spans 900 Hz–5 kHz, extending approximately 600 Hz toward lower frequencies compared with *k* = 0 (with a calculation interval of 50 Hz), and the energy dissipation in the high-frequency range is also substantially enhanced. At *k* = 0, the energy dissipation in rubber1 is concentrated mainly in the 2–3 kHz band, while that in rubber2 is concentrated near 1 kHz and in the 4–5 kHz band, with the overall dissipation level remaining below 0.4 in both layers. At *k* = 80%, the shear-wave energy dissipation in both rubber1 and rubber2 is significantly enhanced, particularly in rubber2 located at the end of the MLM, where the shear-wave energy dissipation increases by approximately 0.21 (in terms of the difference in ${\bar{W}}_{2,S}$), indicating that the acoustic wave is more effectively dissipated after being modulated by the MLM. This enhancement is partly attributable to the MLM strengthening the longitudinal-to-shear wave conversion: the proportion of shear-wave energy dissipation (${\bar{W}}_{2,S}$/${\bar{W}}_{\mathrm{total}}$) at *k* = 80% is approximately 10% higher than that at *k* = 0. The remaining enhancement arises from the reduction in acoustic velocity induced by the MLM, which prolongs the propagation time of acoustic waves within the rubber matrix and thereby increases the opportunity for energy dissipation.

### 4.2. Hybrid MLM for Synergistic Broadband Sound Absorption

**Figure 9:**
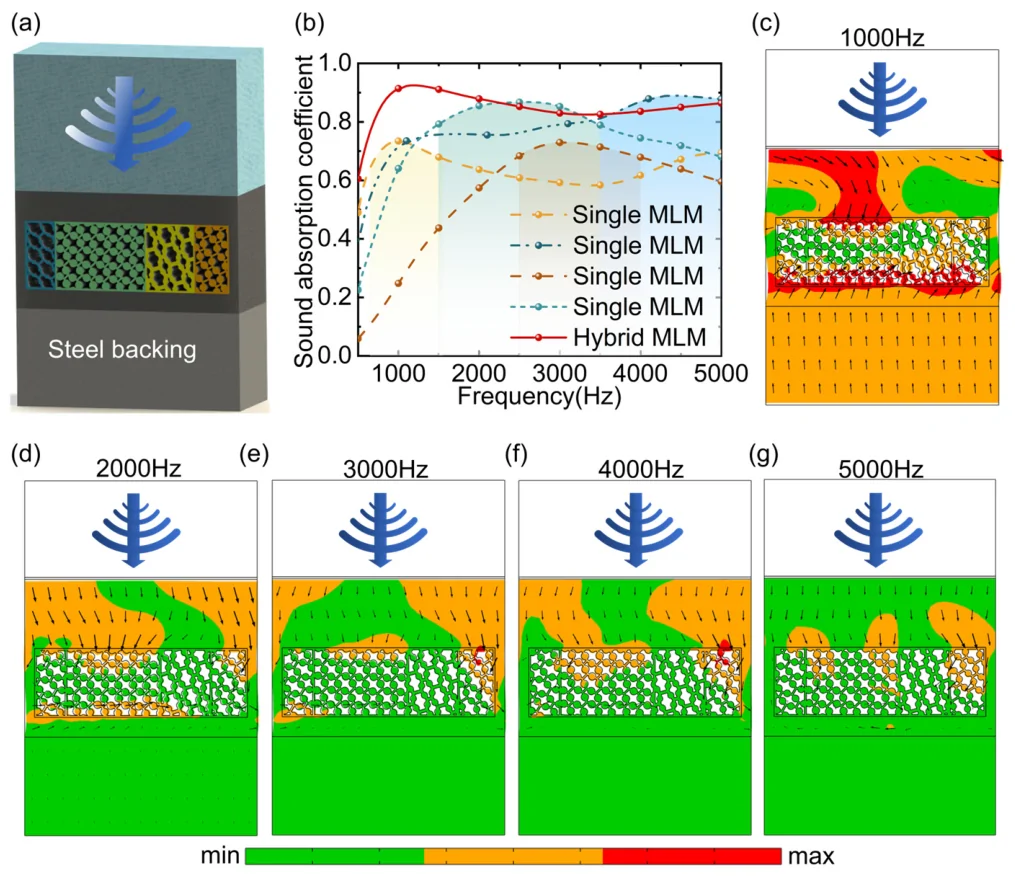
Broadband acoustic absorption characteristics of the hybrid MLM absorption structure. (**a**) Configuration schematic of the rubber–MLM-steel backing composite structure; (**b**) acoustic absorption coefficient spectra of the single structure and the four-grid composite structure (red curve). The composite structure synergistically combines the absorption peaks of the individual structures, yielding broadband acoustic absorption across the 500–5000 Hz band with an average absorption coefficient of 0.85; (**c**–**g**) stress field distribution contours within the structure under acoustic excitation at different frequencies.

MLM units with different wall thicknesses and mass fractions respond to different frequency ranges. To broaden the absorption band, the design domain is divided into four blocks, each with its own MLM geometry (Figure 9a). The geometric parameters are optimized with the overall absorption coefficient as the objective, constrained by the printable range. The resulting hybrid arrangement broadens the absorption through coupling between adjacent units [67,68].

Figure 9b compares the hybrid MLM with the four individual unit structures. The hybrid combines the absorption features of the four configurations and achieves an average coefficient of 0.85 over 500–5000 Hz. Figure 9c–g show the stress distributions at several frequencies. Units with different geometries dominate the response at their corresponding frequencies. The arrows indicate the stress magnitude and direction. After the acoustic wave passes through the MLM, the stress in the rubber is dominated by transverse components, consistent with the preceding analysis.

### 4.3. Sound Absorption Experiment

**Figure 10:**
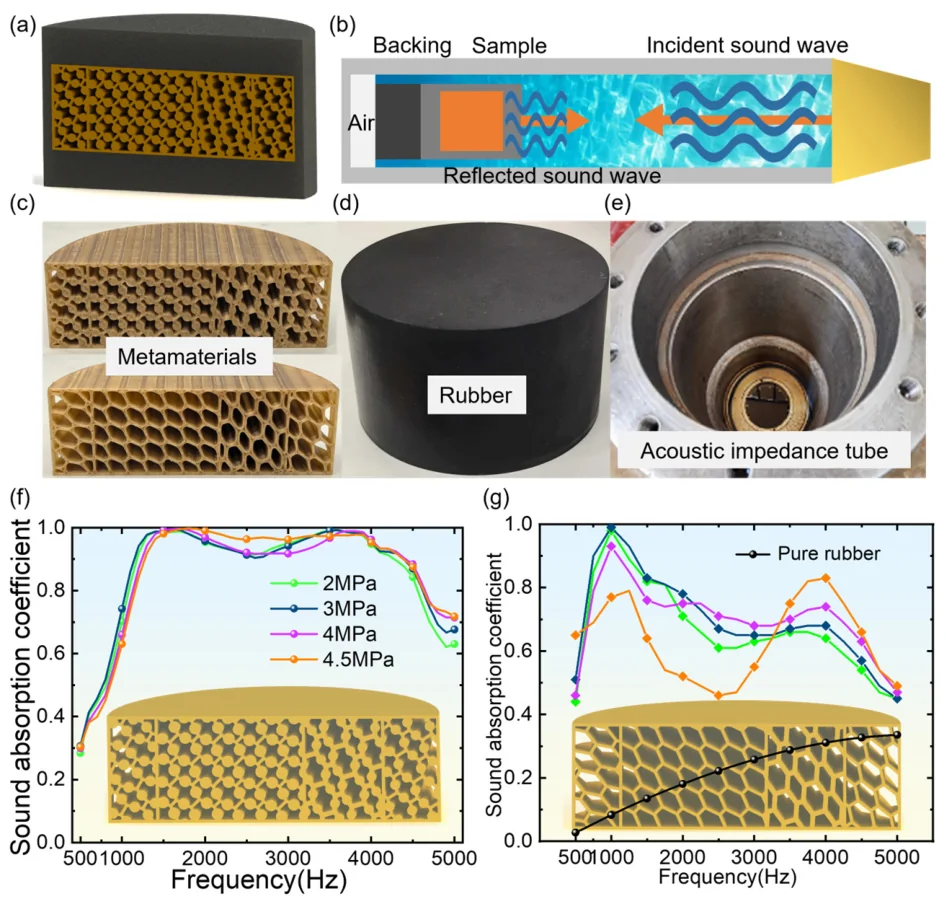
Experimental characterization of the pressure-resistant broadband acoustic absorption performance of MLM. (**a**) Schematic diagram of the composite structure consisting of the hybrid MLM and rubber; (**b**) schematic of the underwater sound absorption measurement setup based on the tube-end method. The composite sample (rubber–MLM-steel backing) is fixed at the end of the impedance tube, while an acoustic transducer at the opposite end emits incident waves. Two hydrophones acquire sound pressure signals for the calculation of the transfer function and absorption coefficient; (**c**) photographs of the MLM sample and the control group physical structures; (**d**) rubber sample for underwater acoustic absorption; (**e**) acoustic impedance tube; (**f**) measured acoustic absorption data of the hybrid MLM; (**g**) measured acoustic absorption data of the structure without mass blocks.

The sound absorption was measured by the impedance tube method. A lossy rubber with a density of 1160 kg/m^3^ was used as the matrix. Its frequency-dependent modulus *E*(*f*) and loss factor *η*(*f*) were measured following Ref. [69] and are given by Equations (13) and (14). Figure 10a shows the cross-section of the hybrid MLM sample: the upper and lower rubber layers are each 20 mm thick, and the middle MLM layer is 30 mm.

(13) $$E(f)=\left[0.402{\left(f/1\mathrm{Hz}\right)}^{0.57558}+4.66\right]\times 10^{6}\;\mathrm{Pa},$$

(14) $$\eta (f)=0.170+0.077\log_{10}\left(f/1\;\mathrm{Hz}\right)+0.145{\left[\log_{10}\left(f/1\;\mathrm{Hz}\right)\right]}^{2}-0.025{\left[\log_{10}\left(f/1\;\mathrm{Hz}\right)\right]}^{3}.$$

Figure 10b shows the setup. The sample was placed at the bottom of the impedance tube, and the absorption coefficient was derived from the reflected wave. A control group with the same wall thickness and rotation angle but without mass blocks was also tested. Figure 10c–e show the fabricated specimens and the tube. Measurements were performed at hydrostatic pressures of 2, 3, 4, and 4.5 MPa (Figure 10f,g).

The MLM achieves an average absorption coefficient above 0.85 over 500–5000 Hz, while the control group absorbs only in narrow bands. Pure rubber of the same total thickness gives an average of 0.22 and a peak of 0.34.

Under hydrostatic pressures up to 4.5 MPa, the absorption performance of the MLM shows no significant degradation, indicating that the MLM maintains its broadband absorption capability under hydrostatic pressure. Table 3 summarizes the quantitative comparison between the control and the MLM at 4.5 MPa. With the mass blocks introduced, the average absorption coefficient rises from 0.61 to 0.87. The bandwidth for α > 0.8 expands from 1800–1900 Hz to 1200–4600 Hz, and a bandwidth of 1300–4300 Hz appears for α > 0.9, whereas the control shows none above this threshold.

## 5. Discussion and Conclusions

This work proposes a mass-loaded metamaterial (MLM) that substantially weakens the inherent modulus–density coupling in lattice materials, reducing the power-law exponent from 2.45 to as low as 0.287, and extends this static decoupling into the dynamic regime, where the modulus increases while the wave velocity decreases. The main contributions are as follows.

First, in terms of mechanical decoupling, the MLM establishes a many-to-many parametric mapping between modulus and density, breaking the one-to-one correspondence inherent in conventional materials.

Second, in terms of wave regulation, the mass-loading design further decouples acoustic velocity from modulus. As the mass-loading ratio increases, the acoustic velocity decreases significantly (a theoretical reduction of 35%, with the downward trend verified experimentally), while the modulus remains nearly unchanged and the acoustic impedance rises accordingly. The metamaterial therefore simultaneously possesses low wave velocity, high modulus, and high impedance, three properties that are mutually exclusive in conventional materials.

Third, in terms of application potential, by integrating unit cells with different mass-loading ratios, the hybrid MLM achieves an average underwater sound absorption coefficient greater than 0.85 over 500–5000 Hz. Under hydrostatic pressures up to 4.5 MPa, the absorption performance shows negligible degradation, confirming its combined capability of pressure resistance and broadband absorption.

The above results indicate that the MLM is an effective strategy for achieving modulus–density decoupling and extending this decoupling into the dynamic regime. However, certain limitations remain.

Regarding the completeness of decoupling, although the MLM reduces the exponent *r* from 1.37 and 2.45 for the baseline lattices to 0.620 and 0.287 for MLM-A and MLM-B, respectively, *r* does not reach zero. The residual positive correlation arises because the added mass blocks inevitably introduce a certain stiffness through their connection to the framework, albeit far less than in conventional materials. Whether more aggressive mass-block layouts or alternative framework topologies can push *r* closer to zero remains an open question.

Regarding the frequency and angular applicability, this study focuses on the 500–5000 Hz range, where the wavelength is much larger than the 10 mm unit cell size. At higher frequencies, where the wavelength becomes comparable to the unit cell size, Bragg scattering and diffraction may degrade the absorption performance and invalidate the homogenization description. In addition, only normal incidence is considered in this study. Under oblique incidence, the propagation paths within the MLM–rubber composite will change, and the mode conversion efficiency is expected to vary with the incident angle. Before practical application, further work is needed, including absorption tests under oblique incidence and diffuse-field conditions, extension to lower and higher frequencies, hydrostatic tests beyond 4.5 MPa, and long-term durability assessment under seawater immersion.

## Figures and Tables

**Table 1:** Power-law exponents of different lattice metamaterials.

Serial Number	*r*	λ	SE(r)	*r*(95%CI)	R^2^
A	1.37	1.15	0.0232	[1.28, 1.38]	0.999
B	2.45	1.50	0.0230	[2.39, 2.50]	0.999
MLM-A	0.620	0.491	0.0229	[0.557, 0.684]	0.994
MLM-B	0.287	0.132	0.0253	[0.227, 0.347]	0.947

**Table 2:** Material properties of PEEK polymer.

Property	Test Standard	Value
Density Tensile strength Flexural modulus	ISO 11357 ISO 527 ISO 178	1300 kg/m^3^ 80 MPa 4 GPa

**Table 3:** Average absorption coefficients over 500–5000 Hz and bandwidths for α > 0.8 and α > 0.9 under a hydrostatic pressure of 4.5 MPa.

Configuration	Average α (500–5000 Hz)	Bandwidth for α > 0.8	Bandwidth for α > 0.9
Lattice Metamaterial	0.61	1800–1900 Hz	none
MLM (Hybrid)	0.87	1200–4600 Hz	1300–4300 Hz

**Figure A1:**
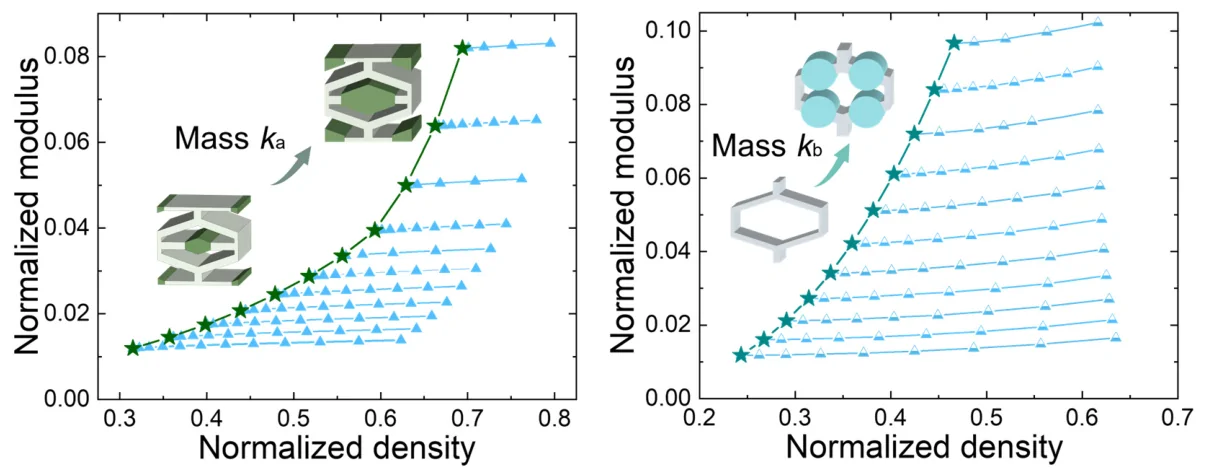
Normalized *x*-direction modulus as a function of normalized density for MLM-A and MLM-B. (**Left panel**) MLM-A. (**Right panel**) MLM-B. Star-marked curves illustrate the modulus–density relationship under varying wall thickness, while triangle-marked curves correspond to the variation induced by adjusting the mass fraction *k*_a_ and *k*_b_. The data clearly reveal a decoupling behavior between the *x*-direction modulus and density.

**Figure A2:**
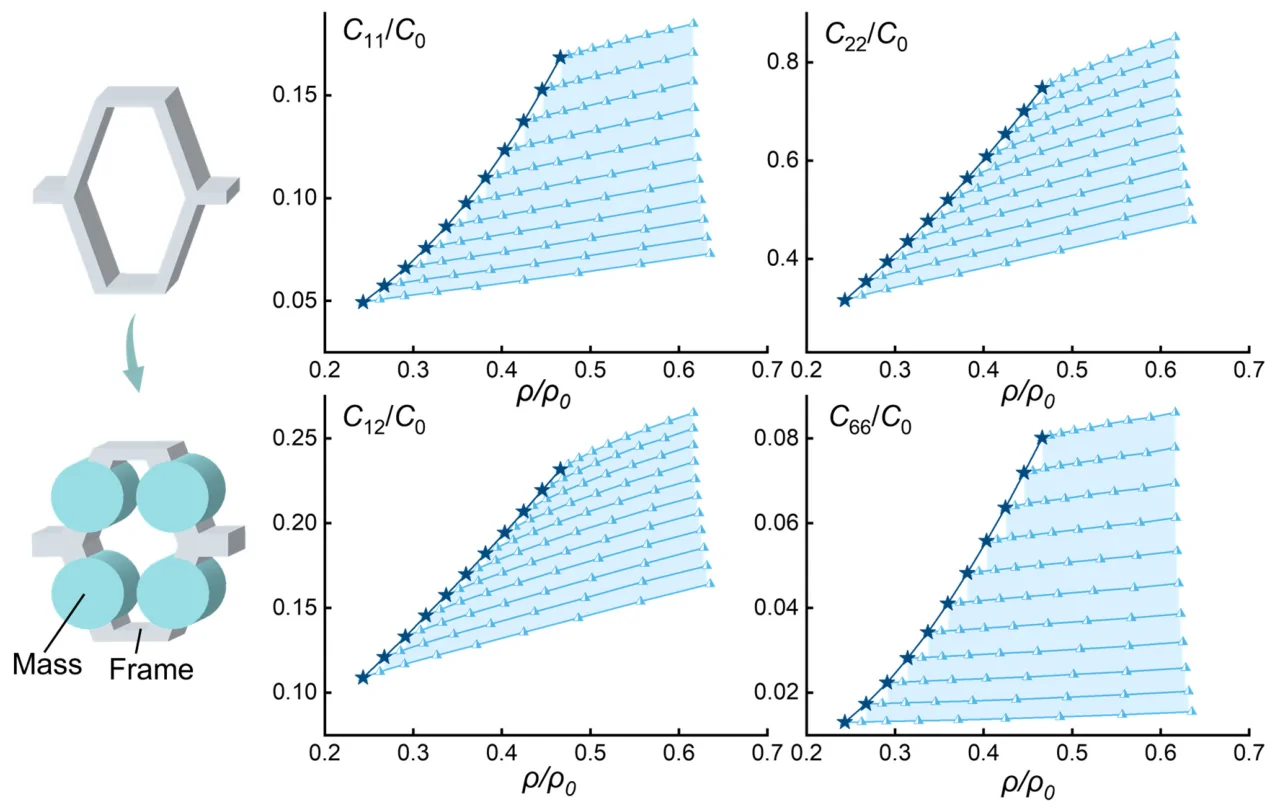
Normalized elastic constants (*C*_ij_/*C*_0_) as a function of normalized density (*ρ*/*ρ*_0_) for the MLM. Panels show the variation in *C*_11_/*C*_0_, *C*_22_/*C*_0_, *C*_12_/*C*_0_, and *C*_66_/*C*_0_. Star-marked curves represent the lattice metamaterial, while the triangle-marked curves represent the MLM. The results demonstrate that the growth rates of all four elastic constants with respect to density are significantly reduced in the MLM, confirming the decoupling characteristics of its elastic matrix parameters.

## Data Availability

The original contributions presented in this study are included in the article and Supplementary Material. Further inquiries can be directed to the corresponding author.

## Supplementary Materials

The following supporting information can be downloaded at: https://www.mdpi.com/article/10.3390/polym18172109/s1, Figure S1: Configuration design and geometric parameters of two types of mass-loaded MLM units; Figure S2: Homogenization principle of the MLM; Figure S3: Schematic diagram of multi-layer configuration for underwater sound-absorption simulation; Table S1: Quantitative comparison of the proposed MLM with recently reported pressure-resistant underwater acoustic absorbers [6,51,70,71]; Table S2: Geometric parameters and design variables of MLM units; Table S3: Printing parameters for all MLM specimens.

## Appendix A

Figure A1 illustrates the variation in the equivalent modulus in the *x*-direction as a function of density. Curves with star markers represent the trend of modulus variation with density when the wall thickness is altered, while those with triangle markers denote the trend when the mass fraction is adjusted. Qualitatively consistent with the *y*-direction trend presented in Figure 3 of the main text, the *x*-direction modulus does not exhibit a significant increase with density, but rather fluctuates within a limited range, indicating that the modulus in this direction also exhibits decoupling characteristics from density.

## Appendix B

Figure A2 presents the decoupling characteristics of the elastic matrix parameters of the MLM. In contrast to the conventional lattice metamaterial, the MLM exhibits substantially reduced growth rates in all four elastic constants ($C_{11}$, $C_{22}$, $C_{12}$, and $C_{66}$) with respect to density, further confirming the effectiveness of the modulus–density decoupling at the stiffness matrix level.
