# Supplementary material for: Mass-Loaded Metamaterials Based on Polymer Matrix with Static–Dynamic Decoupling for Pressure-Resistant Broadband Underwater Sound Absorption
Source: Polymers (Basel). 2026 Aug 30;18(17):2109. doi: 10.3390/polym18172109 (PMC13568000; doi:10.3390/polym18172109)
Supplement: Supplementary file 1 [file polymers-18-02109-s001.zip › polymers-4509732-supplementary.pdf]

Supplementary Material

Supplementary Material S1

Table S1. Quantitative comparison of the proposed MLM with recently reported pressure-resistant underwater acoustic absorbers. The absorption bandwidth is defined as the bandwidth investigated in each original study. The average absorption coefficient is calculated within each structure's own effective bandwidth as reported in the original studies. Density values follow the reporting convention of each original study and are therefore not directly comparable across structures. Structural complexity is classified as low, moderate, or high based on the unit cell geometry, the number of components, and the reported fabrication constraints.

**Indicators**	**Spider-inspired underwater metamaterial**	**Spiral locally resonant**	**Trussed waterborne**	**Impedance-matched composite**	**Proposed MLM**
**Absorption bandwidth**	0.8–4 kHz	0.8–6kHz	0.8–10 kHz	4–20 kHz	0.5–5 kHz
**Average α**	0.87(ambient)<br>0.70(3MPa)	0.54	0.88(ambient)<br>≈0.7(3MPa)	0.903	0.85(4.5Mpa)
**Operating pressure**	3MPa	0.5 MPa	3 MPa	Not tested	4.5 MPa
**Thickness**	50 mm	22 mm	37 mm	69 mm	70 mm
**Density**	Not reported	4600 kg/m³	Not reported	5500 kg/m³	764 kg/m³
**Structural complexity**	High	Moderate	Moderate–high	Moderate	Low–moderate
**Ref.**	[1]	[2]	[3]	[4]	

Based on the comparison in Table R1, the core advantage of the MLM lies in its synergistic balance of pressure resistance, lightweight characteristics, and broadband absorption performance: it maintains an average absorption coefficient of 0.85 under a hydrostatic pressure of 4.5 MPa, with a pressure resistance 50% higher than the best-performing compared structure (3 MPa), while its equivalent density (764 kg/m³)

is only 13.9% of that of the impedance-matched composite (5500 kg/m³) and 16.6% of that of the spiral locally resonant structure (4600 kg/m³). Moreover, the MLM exhibits the lowest structural complexity (low–moderate) among all compared structures and is the only structure in the table that shows no significant degradation in absorption performance under pressures exceeding 3 MPa. This combined performance of "high pressure resistance + low density + broadband absorption + simple configuration" demonstrates the unique value of the MLM in deep-water lightweight sound absorption applications.

Supplementary Material S2

Configuration design and geometric parameters of two types of mass-loaded MLM units

Figure S1 presents the configuration design and geometric parameters of the two types of mass-loaded MLM units. The key design variables adopted in this work are the wall thicknesses l_a and l_b, as well as the dimensionless mass block ratios k_a and k_b. Specifically, k_a and k_b are defined as the ratios of the actual mass block size to the maximum allowable size, with their denominators H and R representing the maximum mass block dimensions corresponding to k_a=1 and k_b=1, respectively. The specific dimensions and variable relationships are summarized in Table S2.

MLM-A unit configuration. As shown in the left panel of Figure. S1(a), the basic geometry of the lattice-type metamaterial unit without a hexagonal mass block is defined by several key parameters, including the unit side length a=10mm, the reference base length d=1.5mm, the wall thickness l_a (variable), and the critical angles (110° and 140°). The middle panel presents the designed basic MLM-A configuration, where the hexagonal mass block is centrally embedded. The size of the hexagonal mass block is characterized by the variable h, and its maximum allowable size is denoted by H. The right panel displays the MLM-A unit with the maximum mass block ratio (k_a=1), demonstrating the design concept of performance regulation by adjusting the mass block ratio.

MLM-B unit configuration. The left panel of Figure. S1(b) shows the geometric design of the lattice unit without circular mass blocks, with core parameters labelled including the unit side length a the reference base length d, the wall thickness l_b (variable), and the critical angle (140°). The middle panel illustrates the MLM-B unit configuration with small-radius mass blocks (r) embedded on the four lateral pillars. The radius r varies from a small value up to a maximum radius R, which is determined by the geometric relationship R=a/2-b, where b=1.5+ $l_b$cos20°+(2.5- l_b/2) tan20°. The right panel shows the optimized configuration with the maximum circular mass block ratio (k_b=1, i.e., r=R), reflecting the design strategy of achieving modulus-density decoupling by varying the size of the mass blocks.

Table S2. Geometric parameters and design variables of MLM units.

Name of Quantity	Value	Description
a	10mm	Lattice size
d	1.5mm	Reference dimension
h	Variable	Size of the similar hexagonal mass block
H	Variable	Maximum size of the similar hexagonal mass block
r	Variable	Radius of the circular mass block
R	$a/2$-b	Maximum radius of the circular mass block
k_a	h/H	Design variable: ratio of hexagonal mass block
k_b	r/R	Design variable: ratio of circular mass block
l_a	Variable	Design variable: wall thickness of MLM-A
l_b	Variable	Design variable: wall thickness of MLM-B
b	1.5+ l_b*cos20°+(2.5- l_b/2) *tan20°	Horizontal coordinate of the circle center

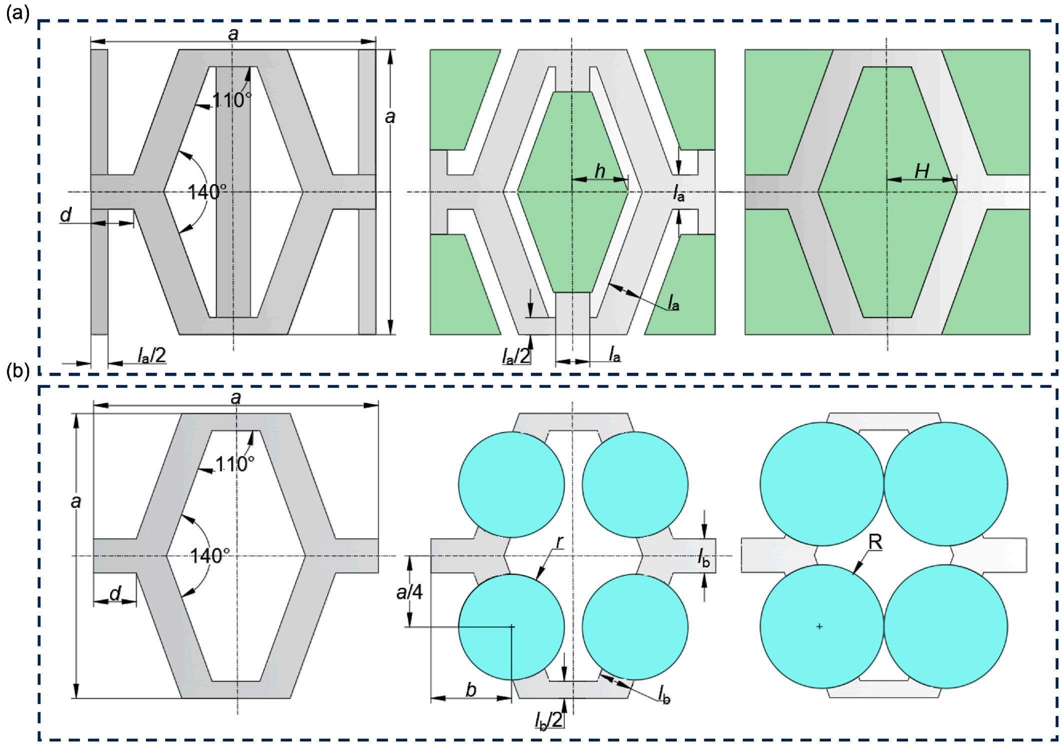

Figure S1. Configuration design and geometric parameters of two types of mass-loaded MLM units. (a) MLM-A unit configuration: The left panel shows the basic geometric design of the lattice-type metamaterial unit without a hexagonal mass block, with key parameters labelled, including unit side length a, base length d, wall thickness la, and critical angles (110°,140°). The middle panel presents the designed basic MLM configuration, while the right panel displays the MLM-A unit configuration with the maximum mass block ratio, demonstrating the design concept of performance regulation by adjusting the mass block ratio of the structure. (b) MLM-B unit configuration: The left panel shows the ge-

ometric design of the lattice unit without circular mass block mounting positions, with core geometric parameters labelled. The middle panel illustrates the MLM-B unit configuration with small-radius (r) mass blocks embedded on the four sides, and the right panel shows the optimized configuration with the maximum circular mass block ratio (radius = R), reflecting the design strategy of achieving modulus-density decoupling by varying the size of the mass blocks.

Supplementary Material S3

Full Derivation of Homogenized Elastic Tensor for Periodic MLM:

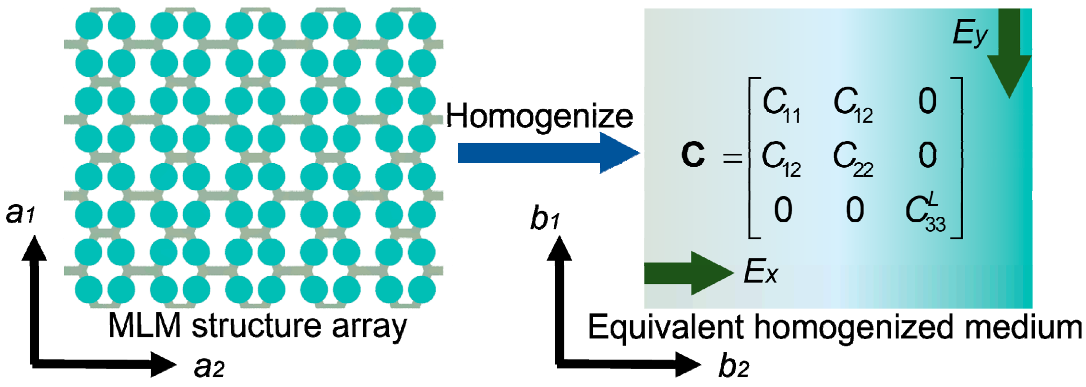

Figure S2. Homogenization principle of the MLM. Schematic illustration of the conversion from the periodic MLM array to an equivalent homogeneous medium via homogenization. The resulting stiffness matrix **C** characterizes the macroscopic mechanical behavior, with the coordinate systems a_1–a_2 and b_1–b_2 denoting the microstructural and macrohomogenized domains, respectively.

1. Two-Scale Decomposition and Asymptotic Expansion of Field Quantities

For two-dimensional periodic MLM underwater acoustic metamaterials, two coordinate systems are introduced to separate scale effects: the macroscopic slow-varying coordinate **b**= (b_1, b_2) describing the global structural domain, and the microscopic fast-varying coordinate **a**= (a_1, a_2) characterizing the internal microgeometry of a single unit cell. A small scale separation parameter $\xi \ll 1$ is defined as the ratio between the characteristic size of the unit cell and the global structural dimension, establishing the mapping relation between the two scales:

$$\mathbf{a} = \frac{\mathbf{b}}{\xi} \tag{S2-1}$$

Based on the chain rule for multivariate composite functions, the spatial differential operator can be decomposed into macroscopic and microscopic partial derivatives:

$$\frac{\partial}{\partial b_i} = \frac{\partial}{\partial b_i} + \frac{1}{\xi}\frac{\partial}{\partial a_i}, \quad i = 1,2 \tag{S2-2}$$

Here, the first term on the right-hand side denotes the partial derivative with respect to the macroscopic coordinate b_i with the microscopic coordinate a_i held fixed, while the second term denotes the partial derivative with respect to the microscopic coordinate a_i with b_i held fixed. The displacement, strain and stress fields inside the metamaterial depend simultaneously on both macroscopic coordinate **b** and periodic microscopic coordinate **a**. Truncating high-order infinitesimal terms, the first-order asymptotic expansion decomposes each physical field into a uniform macroscopic average component and a periodic microscopic perturbation component:

$$\begin{cases} u_i^{\xi}(\mathbf{b}) = u_{0i}(\mathbf{b}) + \xi\chi_i(\mathbf{b},\mathbf{a}), \\ \varepsilon_{ij}^{\xi}(\mathbf{b}) = \varepsilon_{0ij}(\mathbf{b}) + \xi\varepsilon_{ij}^{\chi}(\mathbf{b},\mathbf{a}), \\ \sigma_{ij}^{\xi}(\mathbf{b}) = \sigma_{0ij}(\mathbf{b}) + \xi\sigma_{ij}^{\chi}(\mathbf{b},\mathbf{a}), \end{cases} \tag{S2-3}$$

where u_{0i}, ε_{0ij}, and σ_{0ij} are functions solely of macroscopic coordinate **b,** representing the homogeneous average fields of the equivalent homogenized medium; $\chi_i(\mathbf{b},\mathbf{a})$ denotes the displacement correction function, while ε_{ij}^{χ} and σ_{ij}^{χ} denote perturbation strain and perturbation stress, respectively. All three microscopic fluctuation terms satisfy periodicity over the unit cell domain S, i.e., $\Phi(\mathbf{b},\mathbf{a}+\mathbf{S}) = \Phi(\mathbf{b},\mathbf{a})$.

2. Microscopic Geometric and Constitutive Relations

Under small deformation assumptions, the strain-displacement geometric relation for two-dimensional plane problems reads:

$$\varepsilon_{ij}^{\xi} = \frac{1}{2}\left(\frac{\partial u_i^{\xi}}{\partial b_j} + \frac{\partial u_j^{\xi}}{\partial b_i}\right) \tag{S2-4}$$

Substituting Eqs. (2) and (3) into Eq. (4) and rearranging terms by powers of ξ, the singular ξ^{-1} term automatically vanishes, demonstrating that macroscopic average strain is only determined by the gradient of uniform macroscopic displacement, while microscopic fluctuations solely contribute periodic perturbation strain.

The fourth-order elastic tensor of the matrix material at arbitrary microscopic positions within the unit cell is denoted as $C_{ijkl}(\mathbf{a})$, which varies periodically over the microdomain. Following the Einstein summation convention (i, j, k, l=1,2), the microscopic stress-strain constitutive law follows Hooke's law:

$$\sigma_{ij}^{\xi} = C_{ijkl}(\mathbf{a})\varepsilon_{kl}^{\xi} \tag{S2-5}$$

3. Global Governing Equation from the Principle of Virtual Work

According to the principle of virtual displacements, the internal virtual work equals the external virtual work induced by body forces over the entire metamaterial domain Ω, forming the global balance equation:

$$\int_{\Omega}\sigma_{ij}^{\xi}\delta\varepsilon_{ij}^{\xi}d\Omega=\int_{\Omega}f_i\delta u_i^{\xi}d\Omega \tag{S2-6}$$

where δu_i represents virtual displacement fields constrained by unit-cell periodicity, and f_i denotes microscopic body force components. A core transformation of two-scale homogenization converts the global integral into a macroscopic domain integral superimposed with an area average over the unit cell. Let $|S|$ represent the planar area of a single unit cell; the integral transformation rule is written as:

$$\int_{\Omega}\Psi\left(\mathbf{a}=\frac{\mathbf{b}}{\xi}\right)d\Omega=\int_{\Omega}\left(\frac{1}{|S|}\int_{S}\Psi(\mathbf{a})dS\right)d\mathbf{b} \tag{S2-7}$$

4. Governing Equation for Unit-Cell Microscopic Correction Functions

Fixing the macroscopic uniform strain ε_{0ij} and retaining only periodic perturbation virtual displacements $\delta\chi_i$ to eliminate global macroscopic integrals, the weak-form governing equation for solving displacement correction functions $\chi_k^{mn}(\mathbf{a})$ over the unit cell is derived as:

$$\int_{S}C_{ijkl}(\mathbf{a})\left(\delta_{km}\delta_{ln}+\frac{\partial\chi_k^{mn}}{\partial a_l}\right)\frac{\partial\delta\chi_i}{\partial a_j}dS=0,\quad \forall \text{ periodic virtual displacement } \delta\chi_i \tag{S2-8}$$

where δ_{km} denotes the Kronecker delta. Physically, χ_k^{mn} characterizes the periodic displacement fluctuation field induced inside the unit cell when a unit macroscopic test strain ε_{0mn} is applied.

Periodic displacement boundary conditions are enforced on opposite edges of the unit cell during solution:

$$u(\mathbf{a}_{\text{right}})=u(\mathbf{a}_{\text{left}}),\quad u(\mathbf{a}_{\text{top}})=u(\mathbf{a}_{\text{bottom}}) \tag{S2-9}$$

Two-dimensional plane stress problems contain three independent strain components. Three sets of standard unit test strain tensors are defined for batch computation of microscopic fluctuation fields:

$$\varepsilon^{(1)}=\begin{bmatrix}1&0\\0&0\end{bmatrix},\quad \varepsilon^{(2)}=\begin{bmatrix}0&0\\0&1\end{bmatrix},\quad \varepsilon^{(3)}=\begin{bmatrix}0&1\\1&0\end{bmatrix} \tag{S2-10}$$

Converted into vector form for finite element implementation:

$$\varepsilon^{(1)}=\begin{bmatrix}1\\0\\0\end{bmatrix},\quad \varepsilon^{(2)}=\begin{bmatrix}0\\1\\0\end{bmatrix},\quad \varepsilon^{(3)}=\begin{bmatrix}0\\0\\1\end{bmatrix} \tag{S2-11}$$

5. Finite Element Discretization of the Unit-Cell Problem

Discretize the unit cell into N finite elements. Let S_i denote the area of the i-th element, $\mathbf{B}_i$ the element strain-displacement matrix, and $\mathbf{C}_i$ the microscopic elastic matrix of each element. The global stiffness matrix of the unit cell is assembled by summing elemental stiffness contributions:

$$\mathbf{K} = \sum_{i=1}^{N} \int_{S_i} \mathbf{B}_i^T \mathbf{C}_i \mathbf{B}_i \, dS_i \tag{S2-12}$$

The equivalent nodal load vector corresponding to the n-th group of test strains is constructed as:

$$\mathbf{F}^{(n)} = \sum_{i=1}^{N} \int_{S_i} \mathbf{B}_i^T \mathbf{C}_i \boldsymbol{\varepsilon}^{(n)} dS_i, \quad n = 1,2,3 \tag{S2-13}$$

Without periodic boundary constraints, the raw finite element equilibrium equation for the unit cell takes the form:

$$\mathbf{K}\mathbf{u}^{(n)} = \mathbf{F}^{(n)} \tag{S2-14}$$

A transformation matrix **L** is introduced to condense repeated boundary degrees of freedom and enforce periodic constraints, yielding modified stiffness and load vectors. The final finite element solving equation with periodic boundary conditions is:

$$\mathbf{K}_{\text{per}} = \mathbf{L}^T \mathbf{K} \mathbf{L}, \quad \mathbf{F}_{\text{per}}^{(n)} = \mathbf{L}^T \mathbf{F}^{(n)} \to \mathbf{K}_{\text{per}} \mathbf{u}^{(n)} = \mathbf{F}_{\text{per}}^{(n)} \tag{S2-15}$$

Solving Eq. (S15) sequentially for nodal displacement vectors $\mathbf{u}^{(1)}$, $\mathbf{u}^{(2)}$, $\mathbf{u}^{(3)}$ corresponding to the three test strain groups, the displacement matrix is assembled as:

$$\mathbf{U} = \begin{bmatrix} \mathbf{u}^{(1)} & \mathbf{u}^{(2)} & \mathbf{u}^{(3)} \end{bmatrix} \tag{S2-16}$$

6. Derivation of Macroscopic Homogenized Elastic Tensor

6.1 Continuous Tensor Integral Formulation

Based on strain energy equivalence (the macroscopic average strain energy density equals the area average of microscopic strain energy density over the unit cell), the macroscopic fourth-order homogenized elastic tensor C_{ijkl}^M of the MLM metamaterial is derived from microscopic field integration:

$$C_{ijkl}^M = \frac{1}{|S|} \int_S C_{pqrs}(\mathbf{a}) \left(\delta_{pi}\delta_{qj} + \frac{\partial \chi_{pq}^{ij}}{\partial a_q} \right) \left(\delta_{rk}\delta_{sl} + \frac{\partial \chi_{qr}^{kl}}{\partial a_s} \right) dS \tag{S2-17}$$

The energy consistency verification formula is expressed as:

$$\frac{1}{2} C_{ijkl}^M \varepsilon_{0ij} \varepsilon_{0kl} = \frac{1}{2|S|} \int_S C_{pqrs}(\mathbf{a}) \left(\varepsilon_{0pq} + \varepsilon_{pq}^{\chi} \right) \left(\varepsilon_{0rs} + \varepsilon_{rs}^{\chi} \right) dS \tag{S2-18}$$

6.2 Discrete Matrix Formulation for Finite Element Calculation

Replacing continuous integration with numerical summation over finite elements and substituting the solved displacement matrix **U**, the 3×3 homogenized planar stiffness matrix $\mathbf{C}^{\mathrm{M}}$, directly usable in engineering simulations, is obtained as:

$$\mathbf{C}^M = \frac{1}{|S|} \mathbf{U}^T \mathbf{K} \mathbf{U} \tag{S2-19}$$

7. Calculation of Homogenized Macroscopic Material Properties

Inverting the homogenized stiffness matrix yields the macroscopic compliance matrix $\mathbf{S}^M = \left(\mathbf{C}^M\right)^{-1}$. For plane stress conditions, the equivalent Young's moduli along two orthogonal principal axes are:

$$E_1 = \frac{1}{S_{11}^M}, \quad E_2 = \frac{1}{S_{22}^M} \tag{S2-20}$$

The homogenized mass density of the metamaterial is calculated via area averaging of density distribution over the unit cell:

$$\rho^M = \frac{1}{|S|}\int_S \rho(\mathbf{a})dS \tag{S2-21}$$

Summary of Derivation Logic

The above derivation strictly follows the two-scale asymptotic homogenization framework and consists of three core stages. First, two-scale coordinate separation and asymptotic field expansion distinguish slowly varying macroscopic average fields from fast oscillating microscopic periodic fluctuation fields. Second, the principle of virtual work decouples the multiscale problem to yield a unit-cell boundary value problem for correction functions, which is numerically solved via finite element discretization combined with periodic boundary constraints. Third, the homogenized macroscopic elastic matrix is computed by area averaging microscopic strain energy over the unit cell, from which homogenized Young's moduli, equivalent density and other macroscopic mechanical parameters are extracted. This method converts complex microstructured periodic metamaterials into equivalent homogeneous continua, drastically reducing the computational cost of coupled acoustic-mechanical simulations for large-scale global structures.

Supplementary Material S4

Table S3 Printing parameters for all MLM specimens.

Parameter	Value
Material	PEEK
Infill density	100%

Parameter	Value
Infill pattern	Concentric
Layer thickness	0.2 mm
Nozzle temperature	400–410 °C
Annealing	Not applied (thin-walled specimens, no requirement)
Post-processing	Support removal and surface cleaning only

Note. All specimens within the same comparison group were printed in a single build job with identical parameter settings. The concentric toolpath is the source of the incompletely filled pores near corners and circle centers discussed in the main text and shown in Figure 4(e).

Supplementary Material S5

The numerical model employs a multi-layer stacked configuration, as illustrated in Figure S3. A normally incident plane wave is applied at the top boundary of the water domain and propagates onto the layered structure. From top to bottom, the assembly comprises the water domain, upper rubber layer, metamaterial functional core layer, lower rubber layer, and steel backing.

The thicknesses of the layers are as follows: upper rubber 20 mm, metamaterial core 30 mm, lower rubber 20 mm, and steel backing 50 mm.

The material parameters used in the simulation are summarized in **Figure S3** and detailed below:

- **Water**: density ρ = 1000 kg/m^3, sound velocity c = 1500 m/s.
- **PEEK (metamaterial core)**: density ρ = 1300 kg/m^3, Young's modulus E = 4GPa, Poisson's ratio v = 0.4.
- **Rubber**: density ρ = 1160 kg/m^3, Poisson's ratio v = 0.480. The rubber exhibits frequency-dependent viscoelastic behavior, characterized by the complex modulus. The storage modulus $E(f)$ and loss factor $\eta(f)$ are described by Eqs.(S5-1) and (S5-2), respectively, and the complex modulus $E^*(f)$ is defined by Eq. (S5-3).
- **Steel backing**: density ρ = 7850 kg/m^3, Young's modulus E = 210GPa, Poisson's ratio v = 0.273.

The bottom surface of the steel backing is set as a rigid boundary. The absorption coefficient is calculated from the ratio of the reflected to the incident pressure fields at the top of the water domain.

$$E(f) = [0.402(f/1\text{Hz})^{0.57558} + 4.66] \times 10^{6}\,\text{Pa} \quad \text{(S5-1)}$$

$$\eta(f) = 0.170 + 0.077\log_{10}(f/1\text{Hz}) + 0.145\left[\log_{10}(f/1\text{Hz})\right]^{2} - 0.025\left[\log_{10}(f/1\text{Hz})\right]^{3} \quad \text{(S5-2)}$$

$$E^{*} = E(f) \times (1 + i\eta(f)) \quad \text{(S5-3)}$$

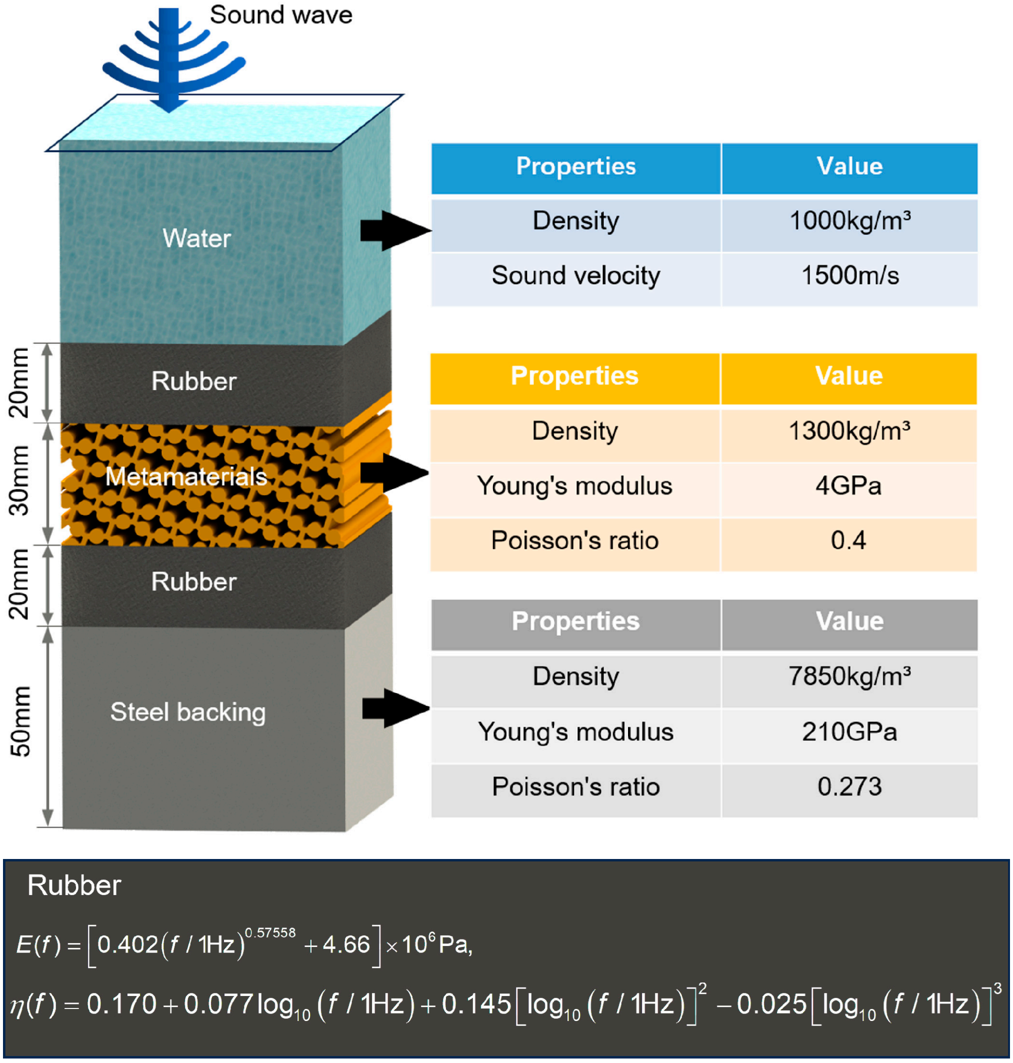

Figure S3. Schematic diagram of multi-layer configuration for underwater sound-absorption simulation. Sound waves are incident from the water domain. The stacked system consists of water, upper rubber layer, metamaterial functional layer, lower rubber layer and steel backing from top to bottom. Material parameters for each medium are listed in corresponding tables. A frequency-dependent complex modulus model is adopted to describe the viscoelastic behaviour of rubber.

Supplementary Material 6

Energy Dissipation Calculation for the MLM–Rubber Composite

1 Fundamental Relationship for Periodic Dissipation in Harmonically Excited Viscoelastic Media

For viscoelastic media subjected to harmonic acoustic excitation, cyclic deformation occurs under periodic acoustic loading. For a viscoelastic system undergoing harmonic deformation at a single frequency, the energy dissipated per cycle is related to the maximum stored elastic energy by:

$$W_{diss} = 2\pi tan\delta \cdot W_{stored,max} \tag{S6-1}$$

where:

- W_{diss} is the total energy dissipated within one vibration cycle;
- $tan\delta$ denotes the material loss tangent;
- $W_{stored,max}$ is the maximum stored elastic energy achieved at the peak deformation instant.

This relation is applied here to each deformation mode separately, which is valid when the longitudinal and shear responses are weakly coupled in the energy sense. When acoustic waves propagate into the acoustic structure, both longitudinal (volumetric) deformation and shear deformation are simultaneously excited. To separate the stored energy and energy dissipation contributed by each deformation mode, the strain tensor obtained from finite-element simulation is decomposed as described below.

The skeleton of the acoustic structure is assumed to be undamped ($tan\delta$=0), so no energy dissipation occurs within the structural skeleton. Energy dissipation is confined exclusively to the two rubber layers.

S6.2 Longitudinal and Shear Elastic Energy Density

From frequency-domain finite-element analysis, the complex strain tensor ε_{ij} of each element is obtained. The strain tensor is decomposed into a volumetric (spherical) component corresponding to longitudinal deformation and a deviatoric component corresponding to shear deformation:

$$\varepsilon_{ij} = \underbrace{\frac{1}{3}\varepsilon_{kk}\delta_{ij}}_{\text{Volumetric (longitudinal) strain}} + \underbrace{e_{ij}}_{\text{Deviatoric (shear) strain}} \tag{S6-2}$$

where ε_{kk} is the trace of the strain tensor, δ_{ij} is the Kronecker delta, and e_{ij} is the deviatoric strain tensor.

Substituting the decomposed strain components into the elastic strain-energy-density formula and evaluating at the peak deformation reached at the end of a quarter period yields the spatial- and frequency-dependent energy-density fields:

- $W_l^{peak}(x, f)$: peak elastic energy density associated with longitudinal (volumetric) deformation, calculated solely from the volumetric strain component.
- $W_S^{peak}(x, f)$: peak elastic energy density associated with shear deformation, calculated solely from the deviatoric strain component.

Note: W_l^{peak} and W_S^{peak} represent instantaneous peak stored-energy densities at maximum deformation, not time-averaged energy densities.

S6.3 Calculation of Dissipated Energy for Individual Deformation Modes

Integrating the peak energy-density field over the spatial domain of each rubber layer gives the maximum total stored elastic energy for that deformation mode within the corresponding layer. Substitution into Eq. (S6-1) yields the energy dissipated per cycle:

$$W_i = 2\pi \tan \delta_i \int_x W_i^{\text{peak}}(x, f)\mathrm{d}x \tag{S6-3}$$

Equation (S6-3) corresponds to Eq. (4) in the main text.

Subscript notation:

- $i=l$ for longitudinal deformation;
- $i=S$ for shear deformation.

$tan\delta_i$ is the loss tangent of rubber for the corresponding deformation mode. For the rubber material used in this work, the loss tangent is assumed to be isotropic, i.e., $\tan \delta_l = \tan \delta_s = \tan \delta$. The subscripts are retained for generality in case future work considers deformation-mode-dependent damping.

The integral $\int_x (\cdot)\mathrm{d}x$ is evaluated over the spatial domain of either rubber1 or rubber2.

Specifically, the four dissipation components are:

1. **Longitudinal deformation in rubber1:**

   $$W_{1,l} = 2\pi \tan \delta \int_{x\in \text{rubber1}} W_l^{\text{peak}} \mathrm{d}x$$

2. **Shear deformation in rubber1:**

   $$W_{1,s} = 2\pi \tan \delta \int_{x\in \text{rubber1}} W_s^{\text{peak}} \mathrm{d}x$$

3. **Longitudinal deformation in rubber2:**

   $$W_{2,l} = 2\pi \tan \delta \int_{x\in \text{rubber2}} W_l^{\text{peak}} \mathrm{d}x$$

4. **Shear deformation in rubber2:**

   $$W_{2,S} = 2\pi \tan \delta \int_{x\in \text{rubber2}} W_S^{\text{peak}} \mathrm{d}x$$

S6.4 Normalized Energy Dissipation

The time-averaged incident acoustic energy flux I is evaluated in the water domain at a plane immediately before the water–rubber interface, ensuring that it represents the true incident power per unit area:

$$I = \frac{1}{T}\int_0^T \mathrm{Re}(p)\mathrm{Re}(v)\mathrm{d}t \tag{S6-4}$$

Where p is the acoustic pressure, v is the particle velocity, and $\mathrm{Re}(\bullet)$ denotes the real-part operator. Normalization against the incident energy flux yields the fraction of incident acoustic energy dissipated by each mode: $\overline{W}_{1,l} = \frac{W_{1,l}}{I}$, $\overline{W}_{1,s} = \frac{W_{1,s}}{I}$, $\overline{W}_{2,l} = \frac{W_{2,l}}{I}$, $\overline{W}_{2,s} = \frac{W_{2,s}}{I}$. The total normalized energy dissipation, plotted as the purple solid curve in Figure 8(d)–(f) of the main text, is the sum of all four dissipation components:

$$\overline{W}_{\mathrm{total}} = \overline{W}_{1,l} + \overline{W}_{1,s} + \overline{W}_{2,l} + \overline{W}_{2,s} \tag{S6-5}$$

SYMBOL	DESCRIPTION
$\boldsymbol{T}$	Period of incident acoustic wave
$\boldsymbol{tan\delta}$	Loss tangent of rubber (assumed isotropic)
W_l^{peak}	Peak elastic energy density for longitudinal deformation
W_S^{peak}	Peak elastic energy density for shear deformation
$\boldsymbol{W_{1,L}, W_{1,S}}$	Energy dissipated per cycle by longitudinal and shear deformation in rubber1
$\boldsymbol{W_{2,L}, W_{2,S}}$	Energy dissipated per cycle by longitudinal and shear deformation in rubber2
$\boldsymbol{P}$	Acoustic pressure
$\boldsymbol{V}$	Particle velocity
$\boldsymbol{I}$	Time-averaged incident acoustic energy flux
$\overline{W}_{1,l}, \overline{W}_{1,S}$	Normalized longitudinal-wave and shear-wave energy dissipation of rubber1
$\overline{W}_{2,l}, \overline{W}_{2,S}$	Normalized longitudinal-wave and shear-wave energy dissipation of rubber2
$\overline{W}_{total}$	Total normalized energy dissipation

Reference

[1] Li H, Li Z, Yang J, Wu J, Chen Y, Wu L, et al. All-in-one underwater quality evaluation metamaterial with mechanical robustness, sound attenuation, and diffuse reflection. Adv Sci 2026;13:e24261. https://doi.org/10.1002/advs.202524261.

[2] Zhong H, Gu Y, Bao B, Wang Q, Wu J. 2D underwater acoustic metamaterials incorporating a combination of particle-filled polyurethane and spiral-based local resonance mechanisms. Composite Structures 2019;220:1–10. https://doi.org/10.1016/j.compstruct.2019.03.091.

[3] Feng J, Liang Q, Yan X, Ren A, Li D. Ultrathin Mechanically Robust Strut-alterable Trussed Waterborne Metamaterial for Broadband Sound Absorption. Adv Funct Materials 2025:e11901. https://doi.org/10.1002/adfm.202511901.

[4] Qu S, Gao N, Tinel A, Morvan B, Romero-García V, Groby J-P, et al. Underwater metamaterial absorber with impedance-matched composite. Sci Adv 2022;8:eabm4206. https://doi.org/10.1126/sciadv.abm4206.
